# A Simulation Study to Compare the Predictive Performance of Survival Neural Networks with Cox Models for Clinical Trial Data

**DOI:** 10.1155/2021/2160322

**Published:** 2021-11-28

**Authors:** Georgios Kantidakis, Elia Biganzoli, Hein Putter, Marta Fiocco

**Affiliations:** ^1^Mathematical Institute Leiden University, Niels Bohrweg 1, 2333 CA Leiden, Netherlands; ^2^Department of Biomedical Data Sciences, Section Medical Statistics, Leiden University Medical Center (LUMC), Albinusdreef 2, 2333 ZA Leiden, Netherlands; ^3^Department of Statistics, European Organisation for Research and Treatment of Cancer (EORTC) Headquarters, Ave E. Mounier 83/11, 1200 Brussels, Belgium; ^4^Department of Clinical Sciences and Community Health & DSRC, University of Milan, Fondazione IRCCS Istituto Nazionale Tumori, Via Giacomo Venezian 1, 20133 Milan, Italy; ^5^Trial and Data Center, Princess Máxima Center for Pediatric Oncology (PMC), Heidelberglaan 25, 3584 CS Utrecht, Netherlands

## Abstract

**Background:**

Studies focusing on prediction models are widespread in medicine. There is a trend in applying machine learning (ML) by medical researchers and clinicians. Over the years, multiple ML algorithms have been adapted to censored data. However, the choice of methodology should be motivated by the real-life data and their complexity. Here, the predictive performance of ML techniques is compared with statistical models in a simple clinical setting (small/moderate sample size and small number of predictors) with Monte-Carlo simulations.

**Methods:**

Synthetic data (250 or 1000 patients) were generated that closely resembled 5 prognostic factors preselected based on a European Osteosarcoma Intergroup study (MRC BO06/EORTC 80931). Comparison was performed between 2 partial logistic artificial neural networks (PLANNs) and Cox models for 20, 40, 61, and 80% censoring. Survival times were generated from a log-normal distribution. Models were contrasted in terms of the C-index, Brier score at 0-5 years, integrated Brier score (IBS) at 5 years, and miscalibration at 2 and 5 years (usually neglected). The endpoint of interest was overall survival.

**Results:**

PLANNs original/extended were tuned based on the IBS at 5 years and the C-index, achieving a slightly better performance with the IBS. Comparison with Cox models showed that PLANNs can reach similar predictive performance on simulated data for most scenarios with respect to the C-index, Brier score, or IBS. However, Cox models were frequently less miscalibrated. Performance was robust in scenario data where censored patients were removed before 2 years or curtailing at 5 years was performed (on training data).

**Conclusion:**

Survival neural networks reached a comparable predictive performance with Cox models but were generally less well calibrated. All in all, researchers should be aware of burdensome aspects of ML techniques such as data preprocessing, tuning of hyperparameters, and computational intensity that render them disadvantageous against conventional regression models in a simple clinical setting.

## 1. Introduction

Survival analysis (also called time-to-event analysis) is used to estimate the lifespan of a particular population under study. The most common problem that survival analysis addresses is right censoring; a form of missing data in which the time to event is not observed due to follow-up interruption before experiencing the event of interest or time limitations such as study termination (administrative censoring). The most popular statistical model (SM) for right censored data in clinical research is the Cox proportional hazards (PH) model [1] which is semiparametric as it makes a parametric assumption regarding the link of the predictors with the hazard function (PH), but it does not prespecify any distribution for the baseline hazard. Parametric regression methods for survival data include for instance models with the exponential, Weibull, or log-normal distribution of survival time [2, 3].

The number of studies that focus on prediction models is rapidly expanding in the medical field. Furthermore, there is an increased interest in applying machine learning (ML) for prediction by medical researchers and clinicians [4]. Several ML algorithms have been developed and adapted to deal with censoring, as indicated in a recent comprehensive survey by Wang et al. in 2019 [5]. The choice of the appropriate methodology should be motivated by the available real-life data and their complexity. SMs usually perform well if the sample size is low/moderate, if there is a small number of variables (low-dimensional setting) with a low signal to noise ratio or when linearity and additivity are the dominant ways that predictors are associated with the outcome. On the other hand, ML techniques may be a better choice if the sample size is large/huge, if there is a large number of variables (high-dimensional setting) with a high signal to noise ratio or when nonlinearity and nonadditivity are expected to be strong [6]. SMs typically operate under a specific set of assumptions such as proportionality of hazards for the Cox model, whereas ML algorithms are data driven (nonparametric) without imposing any restrictions in the data structure.

Artificial neural networks (ANNs) have been one of the most widely used ML techniques in healthcare. Hence, over the years, researchers have adapted them to time-to-event data [7–11]. A popular approach in the literature is that of Biganzoli et al. who proposed a partial logistic artificial neural network (PLANN) for flexible modelling of survival data [9]. The authors specified the time (in intervals) as an input feature in a longitudinally transformed feedforward network with logistic activation and entropy error function to estimate (smoothed) discrete hazards in the output layer for each time interval. A few years later, Lisboa et al. extended PLANN introducing a Bayesian framework that performs automatic relevance determination (PLANN-ARD) [12]. PLANN and PLANN-ARD have been applied several times [13–17]. PLANN methodology has been developed for competing risks (PLANNCR) [18] and has also been employed under a Bayesian regularization framework (PLANNCR-ARD) [19]. Extensions of the PLANN in terms of architecture (i.e., hyperparameters, activation functions, and time interval specification) were recently discussed by Kantidakis et al. [20].

ML techniques are omnipresent in medicine as they can deal with complex data with many observations and different types of predictors (e.g., clinical and molecular) because of their data-driven nature. In the previous study of our group [20], PLANN extended was developed and validated for complex liver transplantation data with a large sample size and within a high-dimensional setting (62294 patients, 97 risk factors). The method was compared with Cox models showing that it can be a useful tool for both prediction and interpretation. However, it is not uncommon to have a small number of patients recruited in clinical trials and a limited set of predictive features, for instance in cancer trials such as head and neck cancer or sarcoma. Even so, there is an expectation by clinicians that ML models may perform better than SMs. Therefore, in this work, the focus is on ML techniques versus SM for noncomplex clinical data to investigate a different real-life setting. A Monte-Carlo simulation study is performed to compare PLANN original or extended [9, 20] with Cox PH models for right censored survival data in terms of prediction. Hereto, real-life clinical data is mimicked to simulate synthetic data (5 predictors and 250 or 1000 observations) and to address different scenarios which are representative of the real disease (bone sarcoma). The dataset originates from a randomized phase III European Osteosarcoma Intergroup (EOI) study that investigated the effect of dose-intense chemotherapy in patients with localised extremity osteosarcoma [21]. The endpoint of interest is overall survival (OS) defined as the time to death from any cause since the date of surgery.

The aims of this manuscript can be summarized as follows: (i) investigation of the role of ML as a competitor of traditional methods for right-censored survival data in a simple setting using simulations (low-dimensional data with linear and additive dependence relations over covariates and time and small to medium sample size), (ii) systematic evaluation of model-predictive performance for two ML techniques (PLANNs) regarding discrimination and calibration for a number of scenarios (different censoring and sample size), (iii) investigation of robustness for PLANN original (Biganzoli et al.) and PLANN extended (Kantidakis et al.) in scenarios with less observations or less information available (due to data truncation), and (iv) practical relevance of findings.

The paper is organized as follows. In “Methods,” details are presented about the clinical trial data and the simulation procedure. Further sections discuss the Cox model and the two ANNs, model training, and how the predictive performance was evaluated in simulated data. “Results” presents neural networks tuned based on different measures, compares the predictive performance of all models, and examines the impact of scenarios for their predictive ability. The article ends with a “Discussion” about findings and advantages/disadvantages of the methods with respect to this particular clinical setting. All analyses were performed in R programming language version 4.0.1 [22].

## 2. Methods

This section is divided into different subsections with the necessary elements of this work. The clinical data and the simulation procedure are presented. Both Cox models and SNNs (PLANN original and PLANN extended) are discussed, and it is described how the models were trained. This extensive section is concluded with the performance measures that were used to evaluate the predictive ability of the models. More technical details are provided in the supplementary material.

### 2.1. Clinical Data and Imputation Technique

Osteosarcoma is the most common primary bone malignancy, and the third most frequent cancer in adolescents (only lymphomas and brain tumours are more prevalent) [23, 24]. In the 1970s, the introduction of adjuvant chemotherapy (administered after surgery) in the treatment of the disease increased survival rates dramatically with a current 5-year overall survival (OS) rate above 65%. There are no significant advances in the treatment of the disease over the last 10+ years. Received dose, dose intensity, and survival of chemotherapy have been investigated without evidence of difference in overall or progression-free survival [21, 25].

For this project, data was collected from a randomized controlled phase III trial of the EOI between 1993 and 2002 that investigated the effect of intensified chemotherapy on the OS of nonmetastatic extremity osteosarcoma patients (MRC BO06/EORTC 80931). Treatment arm was randomly allocated to 497 eligible patients who had no prior chemotherapy before trial entry and were up to 40 years old. Treatment arms included the combination of cisplatin and doxorubicin (conventional or dose intense schedule with identical total doses). Surgery was planned at 6 weeks for both arms. The conventional two drug regimen (regimen C) consisted of six 3-week cycles with surgery planned between cycles 2 and 3. The dose-intense regimen (regimen DI) consisted of six 2-week cycles with surgery planned between cycles 3 and 4. Results of the trial showed no evidence of difference in OS (primary outcome) between the two treatment arms, despite the statistically significant increase in the histological response rate [21].

Five variables were preselected based on clinical reasoning: (a) importance of particular prognostic factors in medical literature regarding osteosarcoma [21, 24, 26], (b) clinical input from Leiden University Medical Center (LUMC). These were the 4 categorical variables: *treatment arm* (regimen C, regimen DI), *sex* (female, male), *histological response* (poor ≤ 90% tumour necrosis or good > 90% tumour necrosis), and *excision of margin* (unknown/incomplete or complete). There was only one continuous variable *age at the date of surgery*. The clinical endpoint was OS defined as the time to death from any cause since the date of surgery. Note that only patients for whom surgery was performed after completing 2 cycles (in conventional arm) or 3 cycles (in dose-intense arm) were included. According to the study protocol, surgery was performed around 6 weeks since randomization in both treatment arms. Nevertheless, for 56 patients, surgery was substantially delayed for more than 90 days due to toxicity or it was never performed (28 patients each, respectively). These were excluded as well as 19 patients that did not fulfil the eligibility criteria or as well as when information was totally missing. Overall, 422 patients were included in the dataset.

Follow-up survival times ranged from 0.16 to 10.31 years with a median follow-up of 5.06 years (95% CI 4.45-5.60) estimated with reverse Kaplan-Meier [27]. There were 161/422 deaths (61.85% censoring). The dataset contained 4% missing data with 355/422 complete cases (84.1%) for the 5 variables. A visual overview of missing values is provided in Additional file 1. More specifically, there were missing values for two categorical variables: *histological response* (51/422, 12.1%) and *excision of margin* (67/422, 15.9%). The missForest algorithm was applied to reconstruct the missing values in order to make full use of the original data avoiding any waste of data (single imputation) [28]. This is a nonparametric imputation method which does not make assumptions about the data structure. A random forest is built for each variable with missing values (1000 trees were used to produce a stable model), testing all possible variable combinations as responses. It is the most exhaustive/accurate random forest algorithm for missing data. Poor *histological response* was imputed 27 times and good 24 times (242 poor vs 180 good in the final dataset). Unknown/incomplete *excision of margin* was imputed once and complete 66 times (49 unknown/incomplete vs 373 complete in the final dataset). The frequencies of the other 2 categorical variables were as follows: *drug regimen* (203 regimen C vs 219 regimen DI), *sex* (164 females vs 258 males). The mean *age at the date of surgery* was 16.15 years (range 3.60–40.85 years).

### 2.2. Simulations

This study is reported based on guidelines for simulation research in healthcare [29, 30]. The simulation procedure was repeated *B* = 1000 times to generate *N*_1_ = 250 or *N*_2_ = 1000 synthetic patients per dataset. Simulated data closely resembled the original osteosarcoma data described in “Clinical Data and Imputation Technique” following a 4-step approach:

(1) Combinations were counted for the 4 categorical variables. As each variable consisted of 2 levels, this led to 2^4^ = 16 unique combinations presented in Table 1. For all combinations, mean and standard deviation were calculated for variable *age*
(2) Data was independently simulated according to the proportion of the occurrence of the 16 combinations in the original dataset. *Age* was sampled from a normal distribution with the mean and standard deviation determined by the combination
(3) Coefficients for the covariates were obtained with a log-normal regression in the original data [31]. These were then used to simulate survival times from a log-normal distribution. Survival time generation can be written as follows:
  (1) $$\begin{array}{c} \log\left(T\right)=\mu +\beta^{T}x+\sigma \varepsilon , \end{array}$$
  where *T* are the simulated survival times, *μ* is the intercept (part of coefficients), *β* is the vector of estimated coefficients for the 5 predictors in the original data, **x** is the covariate matrix for a given simulated dataset, *σ* scale parameter (part of coefficients), and *ε* random error with *ε* ~ *N*(0, 1).
(4) Censoring times were generated with a Weibull distribution [32] with parameters (shape and scale) to create 20%, 40%, 61% (close to original data), or 80% censoring

Initially, censoring times were generated from a Weibull with shape = 2.03 and scale = 5.72 (parameters identified from censoring distribution of the original dataset). This led to simulated datasets with 61% censoring on average. Aiming to investigate the robustness of PLANNs' predictive ability, two (adverse) scenarios were defined with less patients or information on the training data: (i) removing patients censored before the second year or (ii) curtailing patient survival at 5 years (administrative censoring at 5 years). Hereto, a Weibull distribution was used with shape 0.75 (set a priori) and appropriate scale parameter to reach on average 20, 40, 61, or 80% censoring on the simulated datasets (scale parameter 76, 20.5, 6.8, and 2.4, respectively) and at the same time obtain a sufficient number of patients for these extra scenarios (for details, see section 5 of Additional file 3).

For 61% censoring, scenarios 1 (Weibull with shape = 2.03 and scale = 5.72) and 2 (Weibull with shape = 0.75 and scale = 6.8) are presented with details in “Results” and in Additional file 3 (supplementary results). Predictive performance of the methods was not affected by the modification of Weibull parameters for the same censoring percentage. Therefore, it was reasonable to assume (a priori) a shape of 0.75 for the other simulated scenarios.

### 2.3. Cox Proportional Hazards Model

The Cox proportional hazards (PH) regression model is commonly employed to estimate the effect of risk factors in models for time-to-event outcomes on survival outcomes because of its simplicity [1]. This model assumes that each covariate has a multiplicative constant over time effect on the hazard function.

Suppose that data with sample size *n* consist of the independent observations from the triple (*T*, *D*, *X*), i.e., (*t*_1_, *d*_1_, *x*_1_), ⋯, (*t*_*n*_, *d*_*n*_, *x*_*n*_). For the *i*^th^ individual, *t*_*i*_ is the survival time, *d*_*i*_ is the indicator (*d*_*i*_ = 1 if the event occurred and *d*_*i*_ = 0 if an observation is right censored), and *x*_*i*_ = (*x*_1_, ⋯, *x*_*p*_) is the vector of predictors. The hazard function of the Cox model with time-fixed covariates is specified as follows:

(2) $$\begin{array}{c} h\left(t\;|\;X\right)=h_{0}\left(t\right)\exp\left(X^{T}\beta \right), \end{array}$$

where *h*(*t* | *X*) is the hazard at time *t* given predictor values *X*, *h*_0_(*t*) is an arbitrary baseline hazard, and *β* = (*β*_1_, ⋯, *β*_*p*_) is the parameter vector.

The corresponding partial likelihood can be written:

(3) $$\begin{array}{c} L\left(\beta \right)=\overset{D}{\underset{i=1}{\prod }}\frac{\exp\left(\sum_{k=1}^{p}\beta_{k}X_{ik}\right)}{\sum_{j\in R\left(t_{i}\right)}\exp\left(\sum_{k=1}^{p}\beta_{k}X_{jk}\right)}, \end{array}$$

where *D* is the set of failures and *R*(*t*_*i*_) is the risk set at time *t*_*i*_ of all individuals who are still in the study at the time just before time *t*_*i*_. This function is maximised over *β* to estimate the model parameters.

### 2.4. Survival Neural Networks

ANNs were inspired from the human brain activity and more specifically from the neurons that transmit information between different areas of the brain. ANNs have a layered structure based on a collection of units called nodes (or neurons) for each layer. The input layer fetches the signals and passes them to the next layer which is called “hidden” after the application of a nonlinear transformation (activation) function. There might be a stack of hidden layers next to each other that connect with the previous layer and transmit signals towards the output layer. Connections between the artificial neurons of different layers are called edges. Artificial neurons and edges have a weight which adjusts through training increasing or decreasing the strength of each connection's signal. To train the network, a target is defined in the output layer which is the observed outcome for each individual. The simplest form of a feedforward ANN has the input layer, a single hidden layer, and the output layer. Feedforward neural networks, which are also called multilayer perceptrons, utilize a supervised learning technique called backpropagation for training [33, 34].

In the medical field, ANNs are popular ML methods, and therefore, their application has been extended to survival analysis. These are usually called survival neural networks (SNNs). Different approaches have been considered; some model the survival probability *S*(*t*) directly or the unconditional probability of death *F*(*t*) [7, 8, 10] whereas other approaches estimate the conditional hazard *h*(*t*) [9, 11, 12]. For this work, the partial logistic artificial neural network (PLANN) approach was applied as developed originally by Biganzoli et al. [9] and its extensions by Kantidakis et al. [20] for a simple feedforward ANN with one hidden layer. PLANN is a SNN with a single output node (unit) which estimates discrete hazards as conditional probabilities of failure. It can be used for flexible modelling of survival data, as it relaxes the PH assumption in intervals.

To implement this approach, survival times are discretized into a set of *l* = 1, ⋯, *L* nonoverlapping intervals *A*_*l*_ = (*τ*_*l*−1_, *τ*_*l*_], with midpoints *α*_*l*_ (time variable), 0 = *τ*_0_ < *τ*_1_ < ⋯<*τ*_*l*_ a set of predefined time points (usually years), and *l*_*i*_ the last observation interval for subject *i*. Data have to be transformed into a longitudinal format where the time variable is added as part of the input features next to the prognostic factors. On the training set, each subject is repeated for the number of intervals being observed, whereas on the test set each subject is repeated for all time intervals. By adding hidden layers, PLANN naturally models time-dependent interactions and nonlinearities between the prognostic features. Here, without loss of generality, each subject was replicated for a maximum of 8 yearly intervals for the main analyses. The last interval included survival times longer than 7 years (as it was not of interest to specify follow-up times longer than 8 years in separate intervals). Similarly, for supplementary analyses, 4 · 8 = 32 or 2 · 8 = 16 time intervals were defined representing 3- or 6-month periods (no separate intervals for follow-up longer than 8 years).

Activation function of both hidden and output layers is the logistic (sigmoid) function:

(4) $$\begin{array}{c} f\left(\theta \right)=\frac{1}{1+e^{-\theta }}. \end{array}$$

The output node is one large target vector with 0 if the event did not occur and 1 if the event of interest occurred in a specific time interval (due to the necessary data transformation). PLANN provides the discrete conditional probability of failure *𝒫*(*T* ∈ *A*_*l*_ | *T* > *τ*_*l*−1_) for each patient at each time interval. Hence, the hazard *h*_*l*_ = *P*(*τ*_*l*−1_ < *T* ≤ *τ*_*l*_ | *T* > *τ*_*l*−1_) is estimated first in each interval and then the survival probabilities *S*(*t*) = ∏_*l*:*t*_*l*_≤*t*_(1 − *h*_*l*_).

The contribution to the log-likelihood is calculated for the all-interval one is at risk. Following Biganzoli et al. [9], the dependence of hazards can be jointly modelled from the time variable *α*_*l*_ and the vector of covariates **x**_*i*_ using as event indicator *d*_*il*_ (with *d*_*il*_ = 1 in the interval *A*_*l*_ containing the event and *d*_*il*_ = 0, otherwise) for discrete survival data as follows:

(5) $$\begin{array}{c} E=-\overset{n}{\underset{i=1}{\sum }}\overset{l_{i}}{\underset{l=1}{\sum }}\left\{d_{il}\log h_{l}\left(x_{i},\alpha_{l}\right)+\left(1-d_{il}\right)\left[1-\log h_{l}\left(x_{i},\alpha_{l}\right)\right]\right\}, \end{array}$$

where *h*_*l*_(**x**_*i*_, *α*_*l*_) are discrete hazard rates which are estimated by the output values *λ*(**x**_*i*_, *α*_*l*_, *w*) with *w* being the weight matrix. The error (loss) function in equation (5) is summed both over the *n* subjects and the time intervals *l* = 1, ⋯*l*_*i*_ in which the subject *i* is observed. It is equivalent to the crossentropy error function used for binary classification problems. By using this error function in an ANN with no hidden layers and the logistic activation function (equation (4)), a linear logistic regression model is obtained.

The PLANN original model can be mathematically framed as follows:

(6) $$\begin{array}{c} \lambda \left(x_{i},\alpha_{l},w\right)=f\left[w_{0k}^{\prime }+\overset{H}{\underset{h=1}{\sum }}w_{hk}^{\prime }g_{h}\left(w_{0h}+w_{1h}\alpha_{l}+\overset{j=p}{\underset{j=1}{\sum }}w_{\left(j+1\right)h}x_{ij}\right)\right], \end{array}$$

where *j* = 1, 2, ⋯, *J* are the nodes in the input layer, *h* = 1, 2, ⋯, *H* are the nodes in the hidden layer, *k* = 1 is the unique node in the output layer, and *x*_i*j*_ are the *p* elements of covariate vector **x**_*i*_. In addition, *w*_*jh*_ are the weights from the input to the hidden layer, *w*_*hk*_′ are the weights from the hidden to the output layer (*w*_0*h*_, *w*_0*k*_′ are the weights of the bias nodes for the input-hidden and the hidden-output layers, respectively), and *g*_*h*_(·), *f*(·) are the activation (transformation) functions for the hidden and the output layers (*f*(·) is given in equation (4)). In “Evaluation of Predictive Performance,” a prognostic score is defined from equation (6), which is used to construct the C-index for PLANNs.

Extensions of the specification of the PLANN approach were applied as described by Kantidakis et al. [20]. SNNs were tuned investigating two more activation functions for the hidden layer of the rectified linear unit (ReLU):

(7) $$\begin{array}{c} g_{h}\left(\eta \right)=\eta^{+}=\max\left(0,\eta \right), \end{array}$$

which is the most frequently used activation function for ANNs and the hyperbolic tangent (tan*h*):

(8) $$\begin{array}{c} g_{h}\left(\eta \right)=\frac{1-e^{-2\eta }}{1+e^{-2\eta }}. \end{array}$$

These activation functions can be seen as different modulators of the nonlinearity transferred to the hidden layer from the input features. Note that the activation function in the output layer was strictly the logistic (sigmoid) shown in equation (4). The *L* nonoverlapping intervals of the discrete survival times were treated as *L* separate variables (1 + *L* + *p* nodes in the input layer instead of 1 + 1 + *p* for PLANN original). However, the extension of PLANN with 2 hidden layers was not applied due to substantial danger for overfitting in this clinical setting with small data (small to moderate sample size, 5 predictors only).

### 2.5. Model Training

Each simulated dataset was randomly split into 2 complementary parts (50% training, 50% test data) under the same event/censoring proportions. To tune the hyperparameters of SNN (PLANN original or extended), 5-fold crossvalidation was performed with grid search on the training part of a simulated dataset with 1000 synthetic patients according to the censoring rate of interest. Training data was divided into 5 folds. Each time, 4 folds were used to train a model and the remaining fold was used to validate its performance (the same folds were used for PLANN original and extended). This procedure was repeated 5 times to take into account all combinations of folds. The performance of the final models with the hyperparameters selected was assessed on the test sets (for each simulated dataset). Packages of implementation for PLANN original [9] and PLANN extended [20] and technical details such as the choice of tuning parameters are provided in Additional file 2. Parameters were tuned on the training data based either on the C-index [35] or on the integrated Brier score (IBS) at 5 years (time point of major clinical interest) [36]. These measures are described in the next section. All analyses were performed in R programming language version 4.0.1 [22].

### 2.6. Evaluation of Predictive Performance

The predictive performance of the models was assessed in terms of discrimination and calibration. The C-index, the Brier score, the integrated Brier score (IBS), and the miscalibration (in terms of absolute accuracy error) were estimated in the simulated test datasets.

In survival analysis, a well-known measure of model performance is Harrell's C-index [35] as an extension of the concept of the receiver operating characteristic (ROC) area [37]. It measures the proportion of all usable pairs of observations (at least one of them has the event of interest) for which the survival times and model predictions are concordant taking into account censoring. Typically, it takes values between 0.5 to 1 with higher values indicating the higher ability of the model to discriminate well. Nevertheless, good discrimination does not imply good calibration and vice versa.

For a Cox model, the predicted survival time of an individual is longer if the linear prognostic index (PI) defined as *X*^*T*^*β* is lower (opposite ranking). This relationship can then be used to calculate the Harrell's C-index to quantify the ability of the model to discriminate among subjects with different event times [35, 37]. For the PLANN, the equivalent is a nonlinear time-dependent PI defined as *θ* = *w*_0*k*_′ + ∑_*h*=1_^*H*^*w*_*hk*_′*g*_*h*_(*w*_0*h*_ + *w*_1*h*_*α*_*l*_ + ∑_*j*=1_^*j*=*p*^*w*_(*j* + 1)*h*_*x*_*ij*_) in equation (6) inside the logistic (sigmoid) activation function of the output layer *f*(*θ*) (see equation (4)). Therefore, equation (6) can be rewritten as follows:

(9) $$\begin{array}{c} \lambda \left(x_{i},\alpha_{l},w\right)=\frac{1}{1+e^{-\theta }}. \end{array}$$

By solving this equation with respect to *θ*, this nonlinear PI can be estimated as follows:

(10) $$\begin{array}{c} \theta \left(x_{i},\alpha_{l},w\right)=\log\left[\frac{\lambda \left(x_{i},\alpha_{l},w\right)}{1-\lambda \left(x_{i},\alpha_{l},w\right)}\right], \end{array}$$

which is the log-odds ratio of the conditional hazard probabilities.

The nonlinear time-dependent PI in equation (10) depends on the covariates, the time interval, and the weights of the network. A simple nonlinear PI that is not time dependent can be obtained by averaging these indexes over all the time intervals

(11) $$\begin{array}{c} \theta \left(x_{i},w\right)=\frac{\sum_{l=1}^{L}\left(\theta \left(x_{i},\alpha_{l},w\right)\right)}{L}. \end{array}$$

Then, this nonlinear PI was used to calculate the C-index for PLANN original. Similarly, a simple nonlinear PI was obtained for PLANN extended by averaging the time-dependent nonlinear PIs over all intervals.

The C-index provides a rank statistic that is not time dependent. Following van Houwelingen and Le Cessie [38], a time-dependent prediction error [36] is defined as

(12) $$\begin{array}{c} \text{Brier}\left(y,\hat{S}\left(t_{0}\;|\;x\right)\right)={\left(y-\hat{S}\left(t_{0}\;|\;x\right)\right)}^{2}, \end{array}$$

where $\hat{S}\left(t_{0}|x\right)$ is the model-based survival probability of an individual beyond *t*_0_ given the predictor *x* and *y* = 1{*t* > *t*_0_} is the actual observation-ignoring censoring.

To assess the performance in simulated data, censored observations before time *t*_0_ have to be considered. To calculate the Brier score when censored observations are present, Graf et al. proposed the use of inverse probability of censoring weighting [36]. Hence, an estimate of the average prediction error of the prediction model $\hat{S}\left(t|x\right)$ at time *t* = *t*_0_ is

(13) $$\begin{array}{c} \text{Er}{\text{r}}_{\text{score}}\left(\hat{S},t_{0}\right)=\frac{1}{n}\underset{i}{\sum }1\left\{d_{i}=1\vee t_{i}>t_{0}\right\}\frac{\text{score}\left(1\left\{t_{i}>t_{0}\right\},\hat{S}\left(t_{0}\;|\;x_{i}\right)\right)}{\hat{C}\left(\min\left(t_{i}-,t_{0}\right)\;|\;x_{i}\right)}. \end{array}$$

In (13), the term $1/\hat{C}\left(\min\left(t_{i}-,t_{0}\right)|x_{i}\right)$ is a weighting scheme known as inverse probability of censoring weighting (IPCW) and score is the Brier score. It ranges (typically) from 0 to 0.25 with lower values indicating smaller prediction error. The Brier score was calculated at 0–5 years (time period of clinical interest).

An overall measure of prediction error is the integrated Brier score (IBS) which can summarize the prediction error over the whole range up to a time horizon of interest $\int_{0}^{t_{\text{hor}}}\text{Er}r_{\text{score}}\left(\hat{S},t_{0}\right)dt_{0}$ (here, *t*_hor_ = 5 years) [2]. The IBS provides the cumulative prediction error up to *t*_hor_ at all available times (e.g., *t*_0_ = 1, 2, 3, 4, and 5 years). As the Brier score, it ranges (typically) from 0 to 0.25.

Last, the predictive ability of the models was evaluated based on their calibration on the test data, which is usually neglected for ML techniques. Calibration refers to the agreement between observed outcomes and predictions [39, 40]. For each method (Cox model, PLANN original, and PLANN extended), the predicted survival probabilities are estimated and the synthetic clinical data are split into *m* = 4 equally sized groups based on the quantiles of the predicted probabilities. Quantiles were chosen over for instance deciles to avoid any computational issues. Then, the observed survival probabilities are calculated using the Kaplan-Meier (KM) methodology [27]. Miscalibration on test sets for each group is defined as the mean squared error (MSE) of the difference between the observed and the predicted survival probabilities:

(14) $$\begin{array}{c} \text{MSE}\left(t_{0}\right)=\frac{\sum_{m=1}^{4}{\left[S_{KM}^{\left(m\right)}\left(t_{0}\right)-{\hat{S}}^{\left(m\right)}\left(t_{0}\right)\right]}^{2}}{4}, \end{array}$$

at *t*_0_ = 2 and *t*_0_ = 5 years.

## 3. Results

In this section, the findings are presented. The following models were compared: (1) Cox model, (2) PLANN original, and (3) PLANN extended in terms of predictive performance in the simulated data with 5 prognostic factors under different percentages of censoring/sample size per dataset. Additional file 3 provides supplementary results for the scenarios, and extra details are not shown here (e.g., hyperparameters selected for the ML techniques and more tables and plots for predictive performance).

### 3.1. Proportional Hazard Assumption

The PH assumption was tested in the original clinical data (*n* = 422) detailed in “Clinical Data and Imputation Technique.” Plots are provided in Additional file 1. The global test for the Schoenfeld residuals was not significant (*p* value = 0.244), and the Schoenfeld residuals showed random patterns against time with coefficients close to 0 suggesting that the proportionality of hazards is not violated [41]. The individual Schoenfeld test for the 5 variables was only significant for *age since the date of surgery* (*p* value = 0.035). Nevertheless, investigation of the plotted Schoenfeld residual values did not show any systematic divergence for *age* from the straight line with a residual value of 0 (no time-dependent effect, Figure S3 of Additional file 1). Moreover, the linear assumption was examined plotting *age* against the martingale residuals of the null Cox model. The log and square root transformations were tested but did not improve its functional form (see Additional file 1). Nonlinearity for *age* seemed to be small. There was no statistical evidence for interactions between risk factors (all *p* values > 0.10 in the multivariate Cox model). For the rest of the analyses, Cox models without interactions between the 5 predictors or time-dependent effects were considered.

### 3.2. SNNs Tuned with the IBS or C-Index

The hyperparameters selected for PLANN original and PLANN extended are provided in section 2 of Additional file 3. Optimal combinations are reported separately for the IBS at 5 years or the C-index. For PLANN original (2 hyperparameters (node) size and decay), a small size was selected for the majority of scenarios by both performance measures. Nevertheless, a larger decay parameter was suggested in general by the networks tuned for the IBS. For PLANN extended, tuning was performed on a 5-D space for parameters nodesize, dropout rate, learning rate, momentum, and weak class weight (see details in Additional file 2). Three activation functions were tested for the input-hidden layer: the “sigmoid” (logistic), the “relu” (rectified linear unit), and the “tan*h*” (hyperbolic tangent). Overall, “tan*h*” and “relu” provided the best performance on the training data for each scenario (IBS or C-index). Optimal parameters for nodesize, dropout rate, learning rate, or momentum differed between the scenarios. A weak class weight of 1 or 1.05 (small adjustment in favor of the weak class) was generally selected.

The performance of tuned SNNs was compared with either the IBS at 5 years or the C-index. Results for scenario 1 with 61% censoring are presented in Table 2. It can be observed that both SNNs had a slightly better predictive performance tuned for the IBS at 5 years. This pattern was consistent for the other scenarios (Tables S10–S13 in Additional file 3). This might be related with the nature of these neural networks, which both predict conditional hazard (death) probabilities *h*_*l*_ for each time interval in a single output node. Then, survival probabilities can be directly estimated at each interval as *S*(*t*) = ∏_*l*:*t*_*l*_≤*t*_(1 − *h*_*l*_). From the 2 predictive performance measures considered here (to train the networks), IBS was calculated through the model-based survival probabilities of an individual beyond *t*_0_ (equation (13)) whereas the C-index was estimated indirectly after the calculation of a nonlinear PI for each individual (equation (11)). Taking everything into account, the IBS at 5 years seemed to be more reliable than the C-index to tune PLANNs. Hence, in the analyses shown below, optimal combinations for the IBS at 5 years were selected for SNNs (PLANN original and extended).

### 3.3. Comparison of Predictive Performance for the Methods

The simulation procedure was repeated *B* = 1000 times to generate *N*_1_ = 250 or *N*_2_ = 1000 synthetic patients per dataset (50% training and 50% test set). In this section, the 3 methods (Cox model, PLANN original, and PLANN extended) are compared based on different predictive performance measures on test data: (i) Brier score from 0–5 years, (ii) Harrell's C-index, (iii) integrated Brier score (IBS) at 5 years, and (iv) miscalibration at either 2 or 5 years. Measures are detailed in “Evaluation of Predictive Performance.” For the sake of simplicity, the focus is on 61% censoring on average (scenario 1). Plots for the other scenarios: 61% scenario 2 and 20%, 40%, and 80% are included in section 4 of Additional file 3.

**Figure 1:**
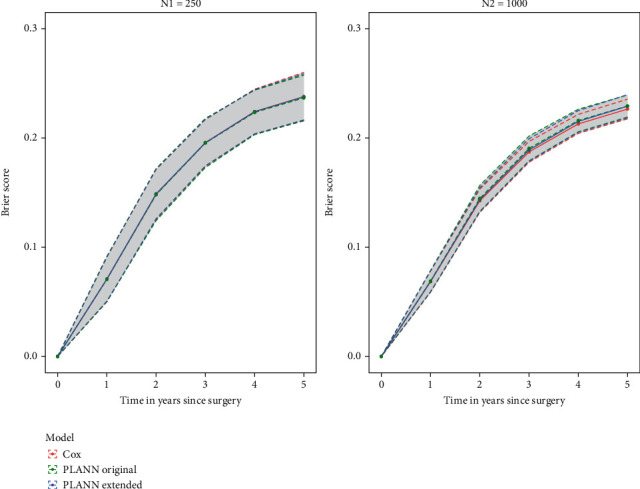
Brier score for Cox, PLANN original, and PLANN extended±one standard deviation for 61% censoring scenario 1. (a) 250 patients; (b) 1000 patients.

Figure 1 shows the Brier score corresponding to each method per year (0–5 years). For a small sample size (*N*_1_ = 250), the performance largely overlapped (the standard deviations (sds) were very similar). For a larger sample size (*N*_2_ = 1000), the Cox model performed slightly better than the SNNs (sd over 1000 datasets was also smaller for the Cox model). For all methods, the predictive performance improved when the sample size increased (smaller Brier scores, higher C-indexes). For 61% censoring scenario 2, results were very similar—especially for a large sample size. For a smaller sample size, PLANN original performed slightly worse than PLANN extended or Cox and had the largest sd. For 80%, censoring results were in the same direction as these. Interestingly, for 20 and 40% censoring PLANN original and PLANN extended performed as good as the Cox model for both sample sizes examined.

**Figure 2:**
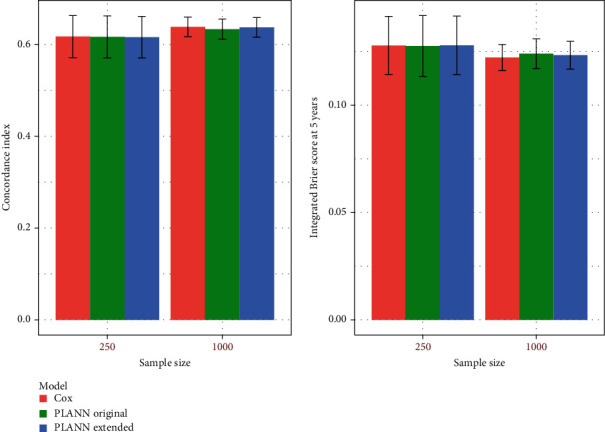
Predictive performance for Cox, PLANN original, and PLANN extended for sample sizes 250 and 1000± one standard deviation for 61% censoring scenario 1. (a) C-index; (b) IBS at 5 years.

The C-index and IBS at 5 years are illustrated in Figure 2 for 61% censoring scenario 1. Regarding the C-index, the performance was very similar for *N*_1_ whereas the Cox model achieved (marginally) the best performance for *N*_2_ very close to PLANN extended. For the IBS at 5 years, the performance was very similar between the methods. The Cox model provided the smallest error for larger sample size (largest sd by PLANN original). For all methods, the performance improved as the sample size increased. Examining the other scenarios, the performance of the methods was very close in terms of the C-index or IBS. Cox models achieved the best performance (and the smallest sds for *N*_2_). For some scenarios (and different sample sizes), PLANN original performed better than PLANN extended and vice versa. This is likely to be related with the optimal parameters selected in each case. The PLANNs fitted might have been out of control for a smaller sample size due to the insufficient amount of regularization implied by the parameters, but the results improved for a larger sample size.

**Figure 3:**
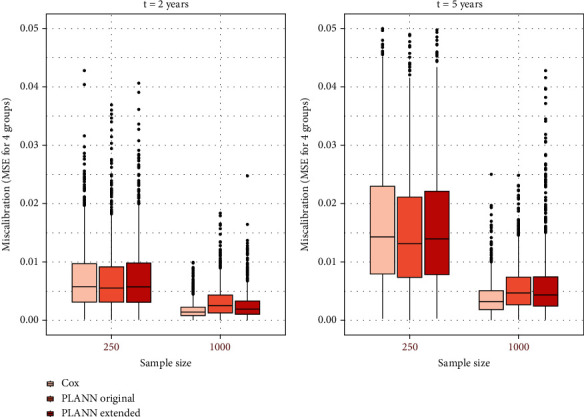
Miscalibration for Cox, PLANN original, and PLANN extended per sample size and 61% censoring (scenario 1). (a) 2 years; (b) 5 years.

Furthermore, the miscalibration of the methods was compared with boxplots in Figure 3 for 61% censoring (scenario 1). For *N*_1_ = 250, PLANN original achieved a slightly better performance at 2 and 5 years. Nevertheless, for *N*_2_ = 1000 patients, the Cox models had by far the lowest miscalibration error (defined as the MSE for 4 groups on test data). PLANN extended showed the highest number of outliers here. Miscalibration error decreased for datasets with larger sample size. For the rest of the scenarios, a similar pattern was observed. For *N*_1_, miscalibration error was almost the same between the methods (at 2 or 5 years) but for larger datasets in size (*N*_2_ = 1000), the Cox model was clearly better calibrated than PLANNs (for 20%, censoring differences were minimal). In all boxplots, PLANN original or extended had more outliers than Cox models for *N*_2_. This indicates that both were less stable than Cox. Moreover, both SNNs were less calibrated for larger percentages of censoring (less events).

In section 7 of Additional file 3, the effect of interval length (3 monthly or 6 monthly intervals) is reported for 61% censoring (scenario 1). The performance of monthly intervals versus yearly intervals was very similar for PLANN original. This is consistent with the absence of relevant time-dependent effects in the simulated datasets, since an increase of the binning over time intervals should improve the predictive performance if such effects were present [42]. For PLANN extended, the performance slightly deteriorated for monthly intervals. This can be explained by the increase in the number of input features. For PLANN extended, the *L* nonoverlapping intervals were treated as *L* separate variables. Therefore, as 3 monthly and 6 monthly intervals corresponded to 32 and 16 variables (versus 8 for yearly intervals), the complexity of the network increased and its predictive ability decreased. A different parametrization of the time intervals into 1 prognostic factor (input feature) as in PLANN original instead of dummy coding for each interval would effectively deal with this issue, if monthly intervals are to be considered.

### 3.4. Impact of Adverse Scenarios for Predictive Ability

To investigate the robustness of the methods, the following 2 scenarios were defined on the training part of the simulated data: (a) removing patients censored before the second year or (b) curtailing patients' survival at 5 years. The number of patients affected by these scenarios for different % of censoring is provided in Additional file 3 section 5 (Tables S14–S18).

**Figure 4:**
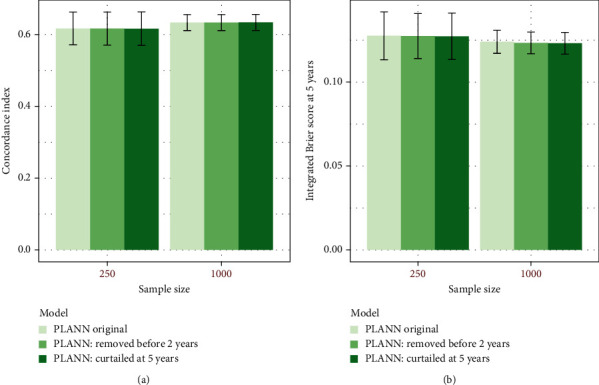
The predictive performance of PLANN original± one standard deviation for sample size 250 or 1000 and 61% censoring (scenario 1). Darker green palette colours correspond to the 2 adverse scenarios (a) removing patients censored before the second year or (b) curtailing patients' survival at 5 years. (a) C-index; (b) IBS at 5 years.

**Figure 5:**
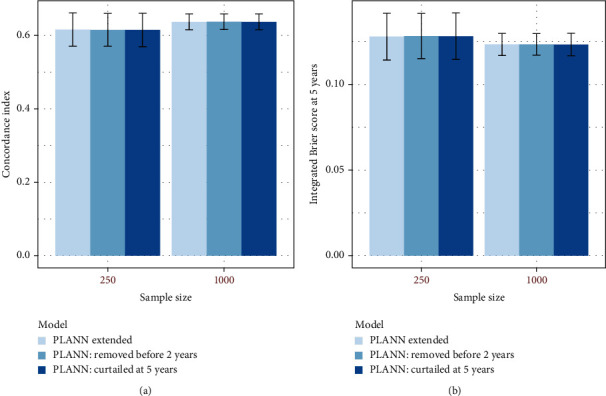
Predictive performance of PLANN extended± one standard deviation for sample size 250 or 1000 and 61% censoring (scenario 1). Darker blue palette colours correspond to the 2 adverse scenarios (a) removing patients censored before the second year or (b) curtailing patients' survival at 5 years. (a) C-index; (b) IBS at 5 years.

Results for PLANN original and PLANN extended for 61% censoring scenario 1 are illustrated in Figures 4 and 5, respectively. The predictive performance for both SNNs did not seem to be affected in terms of the C-index or IBS at 5 years. More plots for SNNs (green and blue palette colours) and the Cox model (red palette colours) can be found in Additional file 3 section 5 for different censoring scenarios. Overall, all methods were quite robust to the adverse scenarios investigated. PLANN extended was less robust than PLANN original for 20% censoring scenario b (administrative censoring) and 80% censoring scenario a (removing patients) for *N*_1_ = 250 (Figures S23 and S29 in Additional file 3).

## 4. Discussion

Nowadays, there is an increased interest in applying ML techniques to create prediction models, because of their intrinsic capability in extracting and modelling the relevant information underlying the available data. This trend is pertinent with the collection of a large volume of patient data in electronic health records (EHR). However, concerns have been raised that the employment of artificial intelligence (AI) for clinical prediction is overhyped in some contexts. Some points of criticism include the use of unsuitable performance measures, overfitting the training data, and the lack of extensive assessment of predictive accuracy (for instance, the absence of calibration curves). Hence, appropriate development/evaluation and transparent reporting of such prediction models is of paramount importance to avoid research waste [43, 44].

Two simulation studies compared PLANN original with Cox models for prediction investigating linear and nonlinear effects for the hazards and several censoring rates [45, 46]. In the first study, Biglarian et al. proposed PLANN for high censoring or when complex interactions are present [45]. In simple models, differences in predictive ability were negligible. Gong et al. found that PLANN is less sensitive to the data size and censoring rates than Cox regression and achieved the best performance when predictor variables assumed nonlinear relationships (or a similar performance elsewhere). ANN extensions of the Cox PH model have been considered as alternatives to PLANN models in the literature for prediction. In 1995, Faraggi and Simon replaced the linear function of the Cox model with the nonlinear output of a feedforward ANN with logistic hidden and linear output layers [47]. Modern deep networks utilize the framework by Faraggi-Simon to extend the Cox model for low- or high-dimensional data [48–50].

In this simulation study, PLANN original and its extensions were compared with traditional regression models in a simple setting with a small to moderate sample size and 5 predictors with synthetic data generated from a clinical trial (MRC BO06/EORTC 80931) in the absence of complex functional dependence relationships involving time and covariates (i.e., nonlinear and nonadditive). Different percentages of censoring and sample sizes were investigated based on well-established performance measures for survival analysis. Both aspects of model discrimination and calibration were evaluated. It was shown that SNNs may reach a comparable performance in terms of the C-index, Brier score, or IBS. The standard deviations (over 1000 repetitions) overlapped to a large extent for all scenarios. Predictive ability was adequately robust to predefined adverse scenarios. However, the Cox models were usually better calibrated (predicted survival probabilities closer to the observed) even though data were not generated from a Cox model. This result in particular shows the relevance of reporting calibration of ML techniques to obtain a neutral comparison with SMs (not reported in the aforementioned articles by Biglarian and Gong). In the paper by Taktak et al. [16], an extensive comparison of different ML models was performed on a large clinical dataset resorting both to discrimination and to calibration measures. The Bayesian extension of the PLANN model (PLANN-ARD) achieved a slightly better performance with respect to the other models. Overall, these results and conclusions are consistent with the present findings, which indicate an urgent need of more attention to model calibration.

Both SNNs were tuned based on global performance measures (IBS at 5 years, C-index) on training data according to the amount of censoring. These measures were chosen as they can summarize the predictive ability of a model in one value, in contrast with the Brier score that is time dependent. For the calculation of the C-index for the PLANNs, it was assumed that there is a monotonic relationship between the predicted survival times and the nonlinear PI obtained (opposite ranking). Such a relationship holds for the Cox model under the PH assumption [37] (between predicted survival times and the linear PI) but is not guaranteed for ML techniques if there are time-dependent effects between the covariates [51]. Nevertheless, the examination of the PH assumption in the original data (from which the data was generated) and the implementation of PLANNs with time coded in 3 monthly or 6 monthly intervals instead of yearly intervals did not improve the performance of the networks (for 61% censoring first scenario) which supports the evidence that no relevant time-dependent effects were present. To explain this, PLANN can estimate complex functional relationships between time and covariates (if present) to improve predictive ability due to the necessary data transformation into a long format with the time split into a set of nonoverlapping intervals. Nonetheless, in the absence of such complex relationships, assuming a monotonic relationship between the predicted survival times and the nonlinear PI is reasonable.

ML techniques such as the SNNs considered in this work have both advantages and disadvantages in the application of the considered clinical data. Some of their most appealing characteristics are the minimal assumptions and the fact that they can model automatically complex (usually high dimensional) data which exhibit nonlinearities and higher order interactions between predictors. Meanwhile, model optimisation is a delicate task requiring robust numerical methods and skillful use. Actually, it should not be neglected that ANNs might converge in suboptimal minima in the error function or not converge on a true and stable local minimum [52], require nontrivial implementation time, and have limited interpretability. More specifically from the two PLANNs examined, PLANN extended required more time and effort for model fine tuning because of the larger number of hyperparameters (5 versus 2 for PLANN original) and the inclusion of time intervals as multiple input features. Therefore, PLANN extended was a more complicated and harder to control network. On the other hand, the standard Cox model makes the PH assumption and implies additivity of effects between the predictors (as any regression model) but offers fast implementation and straightforward interpretation of the estimated coefficients via hazard ratios which is helpful for clinicians to take informed decisions. However, the shape of the hazard function over time can also be extracted from PLANN models allowing for visualisation of their results. An example of this application for breast cancer clinical data is in [14]. Regarding the practical utility in a simple clinical setting, the Cox model has an advantage over ML techniques such as PLANN original or extended. These methods require significantly more resources and time (such as data preprocessing, tuning of parameters, and computational intensity) for merely a comparable predictive performance but also (usually) a suboptimal calibration and a less straightforward interpretation.

The increasing demand for modern methods to improve predictions with survival data has led into the development of several ML algorithms for time-to-event data [5]. Application of such ML techniques should not be pointless but ought to be motivated by exploration of the collected medical data. Building an advanced prediction model powered by AI tools does not necessarily entail a better predictive performance, especially when the sample size and/or the number of features are limited with respect to the complexity of the modeled effects or when the data are not informative enough [6]. A conventional regression model might still provide more accurate survival probabilities and better generalizability on new data in comparison with ML models not developed appropriately to control model complexity. Therefore, in simple clinical settings, ML methods should only be recommended as exploratory tools to assess linear and additive model assumptions.

## 5. Conclusions

Ultimately, the choice of methodology should be based on a combination of factors such as the types of data collected, their size, computational intensity together with the skills in model implementation, and software availability. For this paper, simulated data closely resembled real-life data in a specific clinical setting (low to moderate sample size, small number of predictors) for which the Cox model was expected to be the frontrunner. ML techniques were comparable for a number of suitable predictive performance measures (C-index, Brier score, and IBS) but fall short in terms of calibration. Hereto, there is an urgent need to pay closer attention to calibration (absolute predictive accuracy) of ML techniques to achieve a complete comparison with SMs in medical research. Researchers should also be aware of burdensome aspects of ANNs (data preprocessing, tuning of hyperparameters, and computational intensity), which are not affordable for most nonspecialized researchers that may render them disadvantageous for survival analysis in a simple clinical setting against conventional regression models.

## Figures and Tables

**Table 1:** Proportions for the 16 unique combinations in the original data with 422 patients. Mean and standard deviation of *age at the date of surgery* are provided per combination.

Treatment	Sex	Histological response	Excision	Proportion	Mean age (sd)
Regimen C	Female	Poor	Unknown/incomplete	0.02	12.05 (4.44)
Regimen C	Female	Poor	Complete	0.12	17.04 (7.35)
Regimen C	Female	Good	Unknown/incomplete	0.01	11.06 (2.48)
Regimen C	Female	Good	Complete	0.05	13.72 (5.17)
Regimen C	Male	Poor	Unknown/incomplete	0.01	12.48 (1.30)
Regimen C	Male	Poor	Complete	0.17	16.70 (6.93)
Regimen C	Male	Good	Unknown/incomplete	0.02	14.30 (2.77)
Regimen C	Male	Good	Complete	0.09	16.07 (5.19)
Regimen DI	Female	Poor	Unknown/incomplete	0.01	14.60 (2.33)
Regimen DI	Female	Poor	Complete	0.08	15.35 (6.24)
Regimen DI	Female	Good	Unknown/incomplete	0.01	13.85 (6.12)
Regimen DI	Female	Good	Complete	0.09	14.34 (5.49)
Regimen DI	Male	Poor	Unknown/incomplete	0.03	15.87 (4.04)
Regimen DI	Male	Poor	Complete	0.14	18.54 (6.02)
Regimen DI	Male	Good	Unknown/incomplete	0.01	10.63 (2.98)
Regimen DI	Male	Good	Complete	0.14	17.11 (5.64)

**Table 2:** Performance of PLANN original and PLANN extended tuned for the IBS at 5 years or the C-index for 61% censoring (scenario 1) and 1000 synthetic patients per dataset. The standard deviation (sd) based on 1000 datasets is provided in parentheses.

Measure	PLANN original IBS	PLANN original C-index	PLANN extended IBS	PLANN extended C-index
Brier score 2 years (sd)	0.145 (0.012)	0.146 (0.012)	0.144 (0.011)	0.144 (0.011)
Brier score 5 years (sd)	0.229 (0.010)	0.232 (0.011)	0.229 (0.011)	0.230 (0.010)
IBS 5 years (sd)	0.124 (0.007)	0.125 (0.007)	0.123 (0.006)	0.124 (0.007)
C-index (sd)	0.633 (0.022)	0.628 (0.023)	0.637 (0.021)	0.631 (0.024)
Miscalibration 2 years (sd)	0.003 (0.003)	0.004 (0.003)	0.003 (0.002)	0.003 (0.002)
Miscalibration 5 years (sd)	0.006 (0.004)	0.007 (0.004)	0.008 (0.006)	0.006 (0.006)

## Data Availability

The research data for this project is private. Access to the full dataset of MRC BO06 trial can be requested to MRC Clinical Trials Unit, Institute of Clinical Trial and Methodology, UCL, London, UK. The R-code developed to perform this simulation study is provided in the following GitHub repository https://github.com/GKantidakis/Simulations-SNNs-vs-Cox. There, the reader will also find additional files including (1) the R environment (R objects) to generate all data during this study, (2) a zip file which provides randomly generated synthetic data (*n* = 1000) for 20, 40, 61 (as original, user defined), and 80% censoring, (3) a zip file which is a comprehensive example of how to run the simulations for synthetic data with 61% censoring (as original), and (4) a word document that provides details about the files and a step-by-step tutorial of how to run the R-code.

## Abbreviations

AI: Artificial intelligence
ANN: Artificial neural network
EHR: Electronic health records
EOI: European Osteosarcoma Intergroup
IBS: Integrated Brier score
IPCW: Inverse probability of censoring weighting
KM: Kaplan-Meier
ML: Machine learning
MSE: Mean squared error
OS: Overall survival
LUMC: Leiden University Medical Center
PH: Proportional hazards
PI: Prognostic index
PLANN: Partial logistic artificial neural network
PLANN-ARD: Partial logistic artificial neural network-automatic relevance determination
PLANNCR: Partial logistic artificial neural network for competing risks
PLANNCR-ARD: Partial logistic artificial neural network for competing risks-automatic relevance determination
ROC: Receiver operating characteristic
sd: Standard deviation
SM: Statistical model
SNN: Survival neural network.

## Supplementary Materials

Supplementary 1 Additional file 1: focuses on the original data since surgery. Includes the missing data pattern, the survival and censoring distributions in the original data, and diagnostics for the proportionality of hazard assumption.

Supplementary 2 Additional file 2: provides information about PLANN original and PLANN extended such as the packages of implementation, technical details regarding the choice of tuning parameters, the crossvalidation procedure, and some references for further reading.

Supplementary 3 Additional file 3: contains the analyses for different simulation scenarios not presented in the Results section of this manuscript.
