# Supplementary material for: A Simulation Study to Compare the Predictive Performance of Survival Neural Networks with Cox Models for Clinical Trial Data
Source: Comput Math Methods Med. 2021 Nov 28;2021:2160322. doi: 10.1155/2021/2160322 (PMC8646180; doi:10.1155/2021/2160322)
Supplement: Supplementary 1 — Additional file 1: focuses on the original data since surgery. Includes the missing data pattern, the survival and censoring distributions in the original data, and diagnostics for the proportionality of hazard assumption. [file 2160322.f1.pdf]

# MRC BO06 / EORTC 80931 clinical trial since surgery (422 patients)

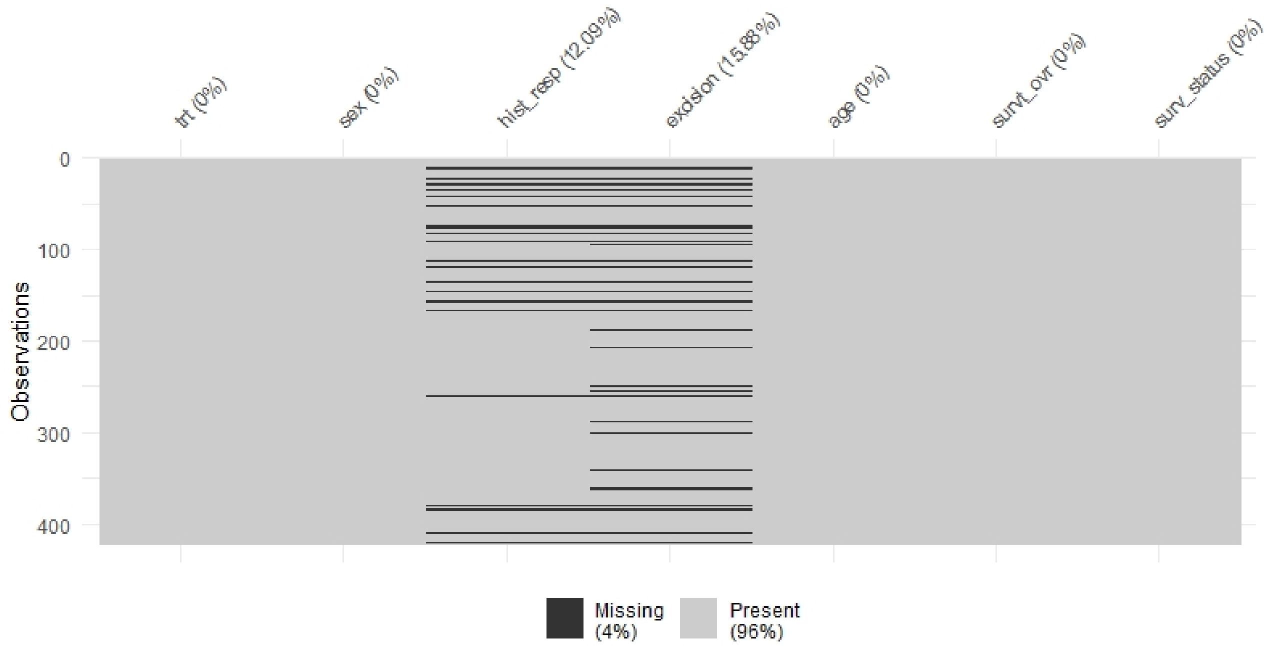


Figure S1: Visual overview of missing values in the original data.

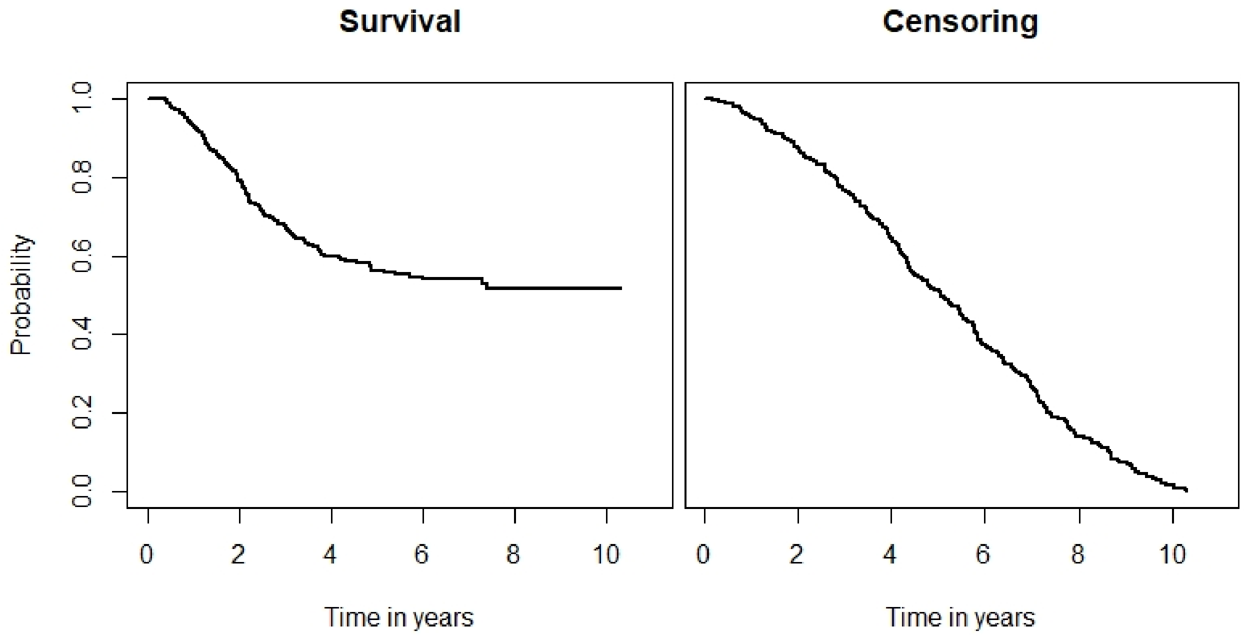


Figure S2: Survival and censoring distribution in the original data.

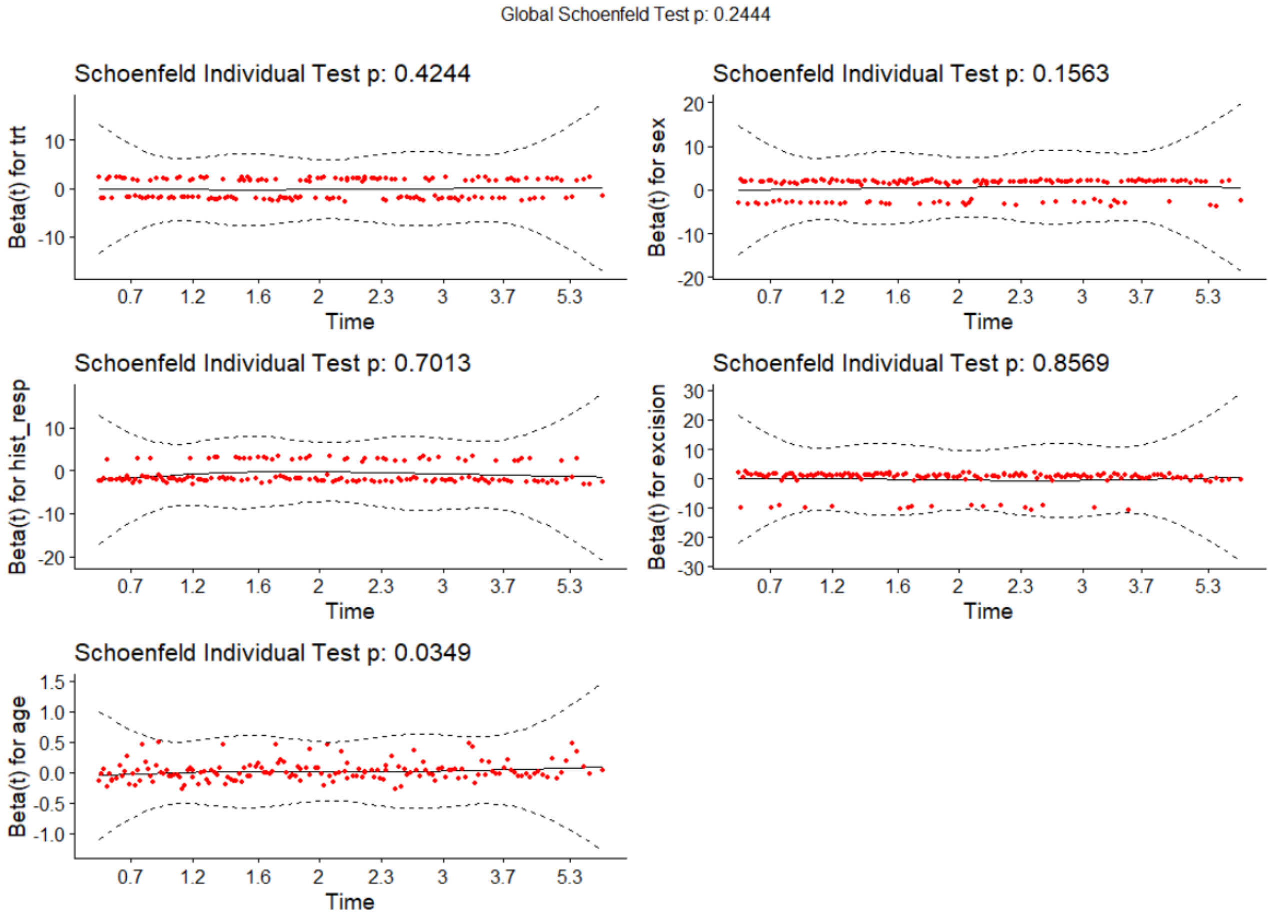


Figure S3: Test the proportional hazards assumption of Cox regression in complete original data (missing values imputed). The global Schoenfeld test indicates that the proportional hazards assumption is not violated for the 5 risk factors.

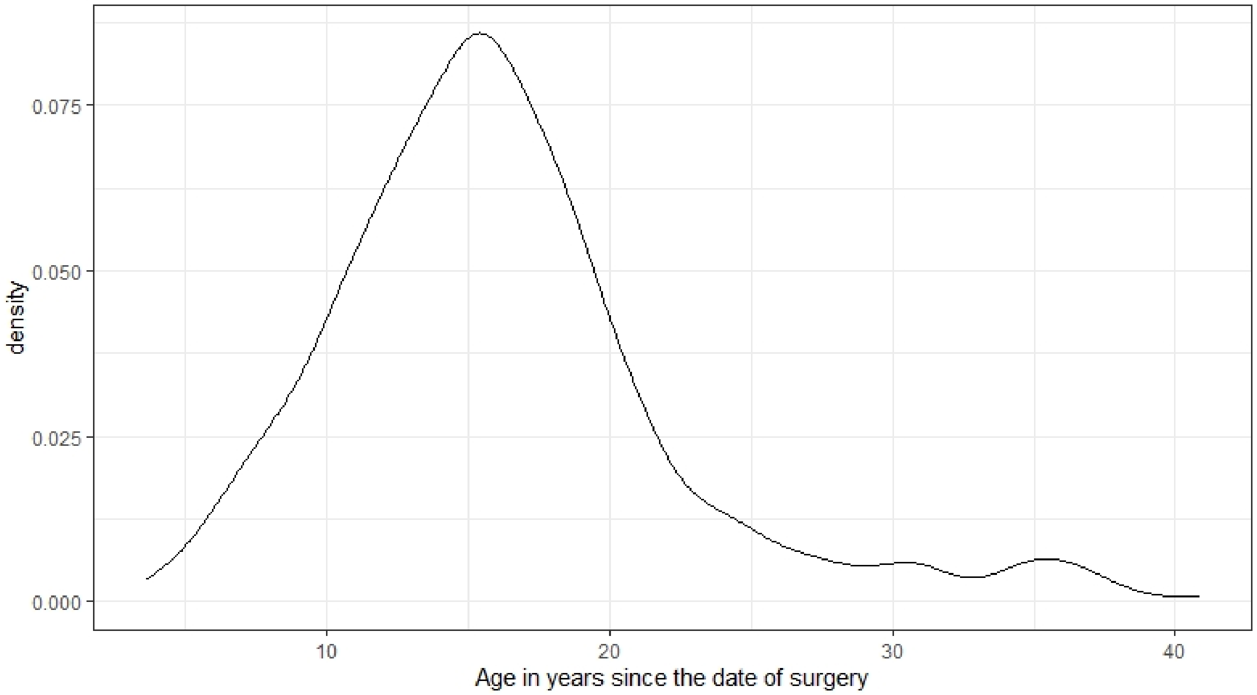


Figure S4: Age of patient in years since the date of surgery in complete original data.

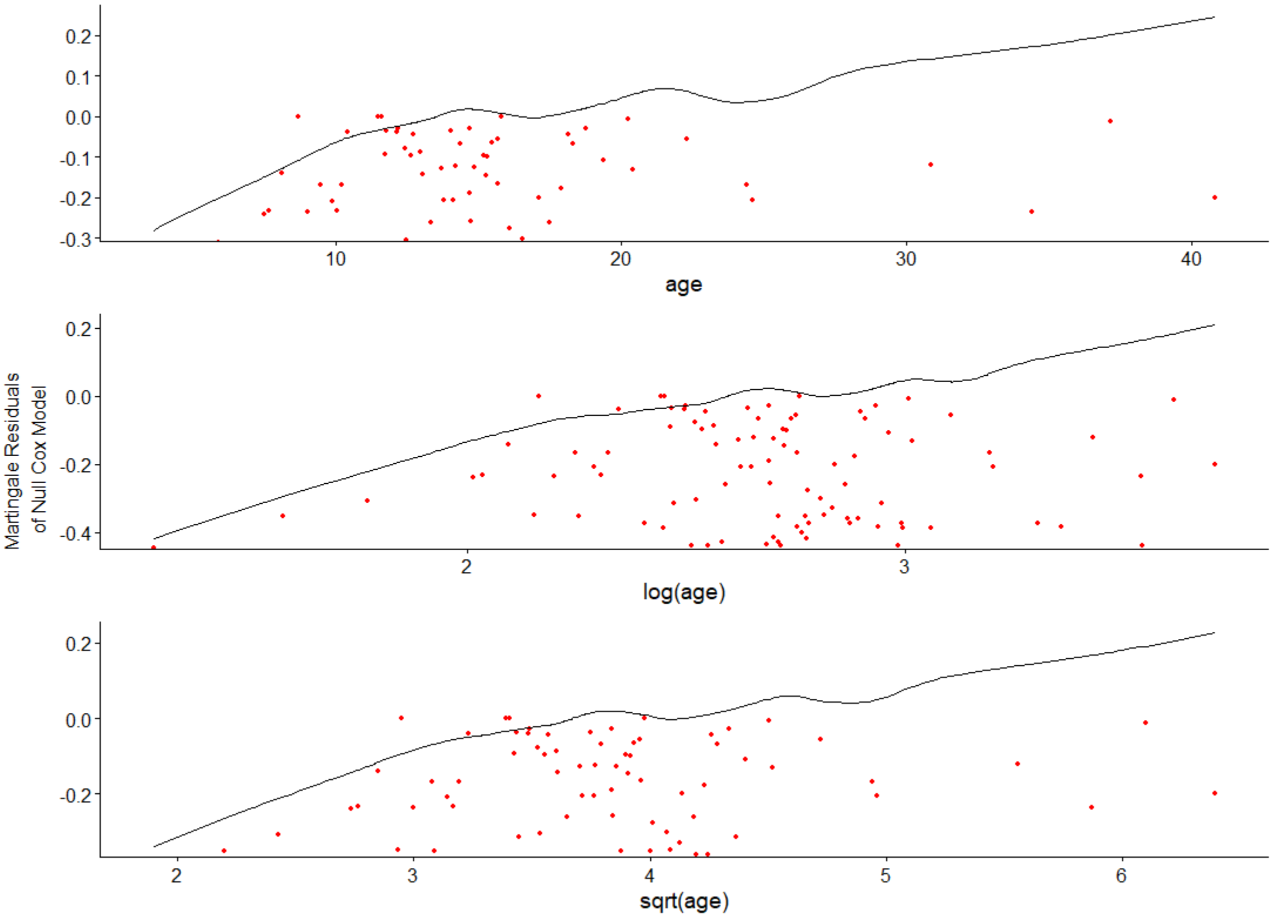


Figure S5: Age is slightly right skewed so the logarithmic and the square root transformations were tested. No difference was observed against the martingale residuals of null Cox proportional hazards model compared to the non-transformed age. Fitted values with lowess (locally weighted scatterplot smoothing) should be linear to satisfy the proportional hazards assumption. Non-linearity seems to be small for age.
