# Supplementary material for: A Simulation Study to Compare the Predictive Performance of Survival Neural Networks with Cox Models for Clinical Trial Data
Source: Comput Math Methods Med. 2021 Nov 28;2021:2160322. doi: 10.1155/2021/2160322 (PMC8646180; doi:10.1155/2021/2160322)
Supplement: Supplementary 2 — Additional file 2: provides information about PLANN original and PLANN extended such as the packages of implementation, technical details regarding the choice of tuning parameters, the crossvalidation procedure, and some references for further reading. [file 2160322.f2.pdf]

# Model training

The split sample approach was employed; simulated data was split randomly into two complementary parts, a training set (50%) and a test set (50%) under the same event/censoring proportions. To tune the hyperparameters of ML algorithms, 5-fold cross validation was performed on the training part of a simulated dataset with 1000 synthetic patients according to the censoring rate of interest. Training data was divided into 5 folds. Each time 4 folds were used to train a model and the remaining fold was used to validate its performance and the procedure was repeated for all combination of folds. Tuning of the hyper-parameters was done using grid search. Performance of final models was assessed on the test sets (of each simulated dataset). Standard software of implementation was not available in R. Therefore, data transformation to longitudinal format was required. The time interval was added next to the other input features to estimate conditional hazard probabilities. Variables were presented in dummy coding - categorical variables as indicators and continuous variables standardized.

For PLANN original, the `nnet` package [1] of the R programming language was used. The choice of hyperparameters and their range (for the grid search) were based on reasoning from the original article by Biganzoli *et al.* [2]. Optimization was done via the BFGS method (quasi-Newton algorithm) of optim R function. Parameters tuned were i) `size` the number of nodes (units) in the hidden layer which determines the number of weights (values 2, 3, $\cdots$, 8) and ii) `decay` a regularization technique applied to the error (loss) function which penalizes large weight values to avoid overfitting as $E^* = E + \lambda \sum w^2$ (values 0.001, 0.005, 0.01, 0.05, 0.1, 0.2, and 0.3). Having 6 inputs in total (5 prognostic variable levels in dummy coding + time interval variable) the optimal value for `size` is somewhere in the range 2-8. The best combination of the parameters on the training data was determined based on either the C-index [3] or the Integrated Brier Score (IBS) at 5 years (time-point of major clinical interest) - a measure that summarises the time-dependent Brier score estimated at different time points until 5 years in one value [4].

For PLANN extended, model tuning was performed in R with the package `keras` [5] which is an interface for the original state-of-the-art neural network library written in Python programming language. `keras` runs on top of `tensorflow` [6] which is a symbolic maths library used for ML. Two of the main advantages of this package are that it allows the use of distributed training of deep learning models on clusters of graphic processing units and the specification of many building blocks such as activation functions, layers, objectives, optimisers. Optimization was done with the stochastic gradient descent algorithm of `keras`. To narrow down the grid of point combinations, search for training data was performed on a 5-D space of some of the most fundamental hyper-parameters. Those are `nodesize` the size of nodes in the hidden layer (values 1, 4, 7, 10, 13, or 16), `dropout rate` that randomly selects the amount of nodes to be dropped-out with a given probability (values 0.1, 0.2 or 0.4), `learning rate` which is the step size of weight iteration (values 0.1, 0.2 or 0.4), `momentum` which helps to accelerate gradient vectors (values 0.8 or 0.9) and `weak class weight` that defines the weight of minority class (values 0.95, 1 or 1.05).

`Nodesize` defines the number of weights of the network and consequently the amount of its complexity. Having 13 inputs in total for the main analyses (5 prognostic variable levels in dummy coding +

8 yearly time intervals), the optimal `nodesize` was somewhere in the range 1 - 16. For supplementary analyses with 3-monthly or 6-monthly intervals, the optimal `nodesize` was in the range 10-40 (5 variables + 32 intervals) or 6-26 (5 variables + 16 intervals), respectively. Therefore, `nodesize` values were adjusted accordingly. `Dropout rate` is a technique used to control over-fitting [7]. Regarding the rest of the parameters, `learning rate` adjusts how fast the stochastic gradient descent iterative method uses stochastic approximation. `momentum` can accelerate the stochastic gradient vectors in the right directions and `weak class weight` can be used for re-weighting unbalanced classes (a small re-weighting adjustment was used). Additionally, we used early stopping to prevent over-fitting (5 epochs tolerance). We specified 50 training epochs (an arbitrary large number) and terminated training once the performance stopped improving on the validation set. The best combinations of hyper-parameters were selected based on either the C-index or the Integrated Brier Score (IBS) at 5 years. Overall, training PLANN extended was more computationally intensive than PLANN original because of the increased number of hyperparameters and input features (the $L$ non-overlapping intervals were treated as $L$ separate variables). Moreover, please note that the implementation of PLANN extended with modern software does not necessarily imply a better performance than PLANN original regarding the numerical optimization.

# References


[1] Brian Ripley and William Venables. nnet: Feed-Forward Neural Networks and Multinomial Log-Linear Models, 2016. URL: https://cran.r-project.org/web/packages/nnet/index.html.

[2] E Biganzoli, P Boracchi, L Mariani, and E Marubini. Feed forward neural networks for the analysis of censored survival data: a partial logistic regression approach. *Statistics in medicine*, 17(10):1169–86, 1998. doi:10.1002/(sici)1097-0258(19980530)17:10<1169::aid-sim796>3.0.co;2-d.

[3] Frank E. Harrell, Kerry L. Lee, and Daniel B. Mark. Multivariable prognostic models: issues in developing models, evaluating assumptions and adequacy, and measuring and reducing errors. *Statistics in Medicine*, 15(4):361–387, 1996. doi:10.1002/(SICI)1097-0258(19960229)15:4<361::AID-SIM168>3.0.CO;2-4.

[4] J. C. van. Houwelingen and Hein. Putter. *Dynamic prediction in clinical survival analysis*. CRC Press, 1st edition, 2012. URL: https://www.crcpress.com/Dynamic-Prediction-in-Clinical-Survival-Analysis/van-Houwelingen-Putter/p/book/9781439835333.

[5] François Chollet. keras, 2015. URL: https://github.com/keras-team/keras.

[6] Martin Abadi, Paul Barham, Jianmin Chen, Zhifeng Chen, Andy Davis, Jeffrey Dean, Matthieu Devin, Sanjay Ghemawat, Geoffrey Irving, Michael Isard, Manjunath Kudlur, Josh Levenberg, Rajat Monga, Sherry Moore, Derek G. Murray, Benoit Steiner, Paul Tucker, Vijay Vasudevan,

Pete Warden, Martin Wicke, Yuan Yu, and Xiaoqiang Zheng. TensorFlow: A system for large-scale machine learning. In *12th {USENIX} Symposium on Operating Systems Design and Implementation ({OSDI} 16)*, pages 265–283, 2016. URL: https://ai.google/research/pubs/pub45381.

[7] Nitish Srivastava, Geoffrey Hinton, Alex Krizhevsky, Ilya Sutskever, and Ruslan Salakhutdinov. Dropout: A Simple Way to Prevent Neural Networks from Overfitting. *The Journal of Machine Learning Research*, 15(1):1929–1958, 2014. URL: http://jmlr.org/papers/v15/srivastava14a.html.
