# Supplementary material for: A Simulation Study to Compare the Predictive Performance of Survival Neural Networks with Cox Models for Clinical Trial Data
Source: Comput Math Methods Med. 2021 Nov 28;2021:2160322. doi: 10.1155/2021/2160322 (PMC8646180; doi:10.1155/2021/2160322)
Supplement: Supplementary 3 — Additional file 3: contains the analyses for different simulation scenarios not presented in the Results section of this manuscript. [file 2160322.f3.docx]

**Additional file 3 – Results**

# About the simulations

B = 1000 simulated datasets with N_1_ = 250 or N_2_ = 1000 patients. Time-to event was generated from a log-normal distribution with estimated parameters from the original data (see table 1 below).

The different simulated scenarios are:

1. Synthetic data with average censoring 20%; censoring was generated from a Weibull (shape = 0.75, scale = 76).
2. Synthetic data with average censoring 40%; censoring was generated from a Weibull (shape = 0.75, scale = 20.5).
3. Synthetic data with average censoring 61%; censoring was generated from a Weibull (shape = 2.03, scale = 5.72). This scenario is called 61% censoring-1 (scenario 1).
4. Synthetic data with average censoring 61%; censoring was generated from a Weibull (shape = 0.75, scale = 6.8). This scenario is called 61% censoring-2 (scenario 2).
5. Synthetic data with average censoring 80%; censoring was generated from a Weibull (shape = 0.75, scale = 2.4).

| **Parameter** | **Estimate** | **Standard error** | **p-value** |
| --- | --- | --- | --- |
| Intercept (μ) | 1.75 | 0.30 | <0.01 |
| Treatment:  Regimen-DI | 0.08 | 0.15 | 0.60 |
| Sex: Male | -0.45 | 0.16 | <0.01 |
| Histological response:  Good | 0.80 | 0.16 | <0.01 |
| Excision: Complete | 0.22 | 0.22 | 0.32 |
| Age | -0.01 | 0.01 | 0.40 |
| Log-Scale (log[σ]) | 0.22 | 0.05 | <0.01 |

Table S1: Log-normal regression on the original data (n = 422). Parameters estimated were used to simulate survival times for synthetic data from a log-normal distribution.

| **N_1_ = 250** | Min. | 1^st^ Qu. | Median | Mean | 3^rd^ Qu. | Max. |
| --- | --- | --- | --- | --- | --- | --- |
| Average 20% | 13.6 | 18.4 | 20.0 | 20.1 | 22.0 | 27.2 |
| Average 40% | 30.8 | 37.6 | 40.0 | 40.0 | 42.4 | 51.2 |
| Average 61% -1 | 50.4 | 58.8 | 60.8 | 60.9 | 62.8 | 70.8 |
| Average 61% -2 | 51.6 | 59.2 | 61.2 | 61.3 | 63.6 | 71.2 |
| Average 80% | 73.0 | 78.3 | 79.7 | 79.8 | 81.3 | 87.0 |

Table S2: Percentages of censoring for 1000 simulated datasets of 250 patients.

| **N_2_ = 1000** | Min. | 1^st^ Qu. | Median | Mean | 3^rd^ Qu. | Max. |
| --- | --- | --- | --- | --- | --- | --- |
| Average 20% | 16.4 | 19.3 | 20.1 | 20.2 | 21.0 | 24.6 |
| Average 40% | 35.2 | 38.9 | 40.0 | 40.0 | 41.1 | 45.8 |
| Average 61% -1 | 56.5 | 60.0 | 61.1 | 61.1 | 62.2 | 66.1 |
| Average 61% -2 | 56.6 | 60.2 | 61.4 | 61.3 | 62.3 | 66.2 |
| Average 80% | 75.3 | 79.0 | 79.8 | 79.8 | 80.7 | 83.5 |

Table S3: Percentages of censoring for 1000 simulated datasets of 1000 patients.

# Hyperparameters selected for SNN

## Hyperparameter selection for PLANN original tuned with IBS and C-index from a random synthetic dataset with N = 1000.

Optimal combinations for IBS were used in the final models presented in the manuscript and in this additional file, as they provided in general better performance in terms of IBS and C-index compared to optimal parameters based on C-index in the simulated data (see next section for a comparison between IBS and C-index).

| Optimal combination for average censoring 20% | Size | Decay |
| --- | --- | --- |
| IBS | 3 | 0.3 |
| C-index | 2 | 0.001 |
| Optimal combination for average censoring 40% |  |  |
| IBS | 2 | 0.005 |
| C-index | 2 | 0.005 |
| Optimal combination for average censoring 61% - 1 |  |  |
| IBS | 7 | 0.3 |
| C-index | 3 | 0.1 |
| Optimal combination for average censoring 61% - 2 |  |  |
| IBS | 2 | 0.3 |
| C-index | 5 | 0.3 |
| Optimal combination for average censoring 80% |  |  |
| IBS | 3 | 0.3 |
| C-index | 2 | 0.05 |

Table S4: Hyperparameters selected for PLANN original.

## Hyperparameters for PLANN extended tuned with IBS and C-index from the same synthetic dataset as PLANN original with N = 1000.

Activation functions investigated for the input-hidden layer are the logistic (sigmoid), the rectified linear unit (relu) and the hyperbolic tangent (tanh). Best performance per measure is provided in black. Optimal combinations for IBS were used in the final models presented in the manuscript and in this additional file, as they provided in general better performance in terms of IBS and C-index compared to optimal parameters based on C-index in the simulated data (see next section for a comparison between IBS and C-index).

| Optimal combination for average censoring 20% | Node size | Dropout rate | Learning rate | Momentum | Weak class weight |
| --- | --- | --- | --- | --- | --- |
| IBS – sigmoid | 7 | 0.1 | 0.2 | 0.8 | 1.05 |
| **IBS – relu** | 7 | 0.2 | 0.1 | 0.8 | 1 |
| IBS – tanh | 10 | 0.4 | 0.4 | 0.8 | 1.05 |
| **Cindex - sigmoid** | 4 | 0.1 | 0.1 | 0.8 | 1.05 |
| Cindex - relu | 7 | 0.2 | 0.4 | 0.8 | 1.05 |
| Cindex - tanh | 13 | 0.2 | 0.4 | 0.9 | 1 |

Table S5: Hyperparameters selected for PLANN extended and 20% censoring.

| Optimal combination for average censoring 40% | Node size | Dropout rate | Learning rate | Momentum | Weak class weight |
| --- | --- | --- | --- | --- | --- |
| IBS – sigmoid | 4 | 0.1 | 0.1 | 0.9 | 0.95 |
| **IBS – relu** | 4 | 0.2 | 0.2 | 0.9 | 0.95 |
| IBS – tanh | 4 | 0.2 | 0.4 | 0.9 | 1.05 |
| Cindex - sigmoid | 4 | 0.1 | 0.1 | 0.8 | 0.95 |
| **Cindex - relu** | 4 | 0.1 | 0.2 | 0.8 | 1 |
| Cindex - tanh | 13 | 0.1 | 0.4 | 0.9 | 0.95 |

Table S6: Hyperparameters selected for PLANN extended and 40% censoring.

| Optimal combination for average censoring 61% -1 | Node size | Dropout rate | Learning rate | Momentum | Weak class weight |
| --- | --- | --- | --- | --- | --- |
| IBS – sigmoid | 16 | 0.1 | 0.4 | 0.9 | 1.05 |
| IBS – relu | 10 | 0.1 | 0.2 | 0.9 | 1 |
| **IBS – tanh** | 16 | 0.1 | 0.2 | 0.8 | 1.05 |
| Cindex - sigmoid | 7 | 0.2 | 0.4 | 0.9 | 1.05 |
| **Cindex - relu** | 13 | 0.1 | 0.2 | 0.9 | 1 |
| Cindex - tanh | 13 | 0.1 | 0.1 | 0.8 | 1.05 |

Table S7: Hyperparameters selected for PLANN extended and 61% censoring-1.

| Optimal combination for average censoring 61% -2 | Node size | Dropout rate | Learning rate | Momentum | Weak class weight |
| --- | --- | --- | --- | --- | --- |
| IBS – sigmoid | 4 | 0.1 | 0.2 | 0.9 | 0.95 |
| IBS – relu | 7 | 0.2 | 0.2 | 0.8 | 1 |
| **IBS – tanh** | 13 | 0.4 | 0.1 | 0.9 | 1 |
| Cindex - sigmoid | 4 | 0.2 | 0.2 | 0.8 | 1 |
| **Cindex - relu** | 4 | 0.2 | 0.4 | 0.9 | 1.05 |
| Cindex - tanh | 4 | 0.1 | 0.4 | 0.9 | 0.95 |

Table S8: Hyperparameters selected for PLANN extended and 61% censoring-2.

| Optimal combination for average censoring 80% | Node size | Dropout rate | Learning rate | Momentum | Weak class weight |
| --- | --- | --- | --- | --- | --- |
| IBS – sigmoid | 13 | 0.1 | 0.4 | 0.9 | 1 |
| IBS – relu | 10 | 0.1 | 0.1 | 0.8 | 0.95 |
| **IBS – tanh** | 10 | 0.1 | 0.1 | 0.9 | 1 |
| Cindex - sigmoid | 10 | 0.2 | 0.4 | 0.8 | 1.05 |
| **Cindex - relu** | 10 | 0.4 | 0.4 | 0.9 | 1 |
| Cindex - tanh | 13 | 0.4 | 0.4 | 0.9 | 0.95 |

Table S9: Hyperparameters selected for PLANN extended and 80% censoring.

# Predictive performance of SNN: tuned parameters with IBS vs C-index

## For average censoring 61% -2

| **N_2_ = 1000** | **PLANN original**  **IBS** | **PLANN original**  **C-index** | **PLANN extended**  **IBS** | **PLANN extended**  **C-index** |
| --- | --- | --- | --- | --- |
| Brier 2 years (sd) | 0.145 (0.014) | 0.147 (0.014) | 0.145 (0.013) | 0.146 (0.013) |
| Brier 5 years (sd) | 0.232 (0.011) | 0.232 (0.012) | 0.231 (0.012) | 0.235 (0.011) |
| IBS 5 years (sd) | 0.125 (0.008) | 0.130 (0.008) | 0.124 (0.008) | 0.126 (0.008) |
| C-index (sd) | 0.635 (0.023) | 0.631 (0.023) | 0.634 (0.024) | 0.617 (0.065) |
| Miscalibration  2 years (sd) | 0.004 (0.003) | 0.005 (0.004) | 0.004 (0.003) | 0.004 (0.004) |
| Miscalibration  5 years (sd) | 0.009 (0.006) | 0.009 (0.006) | 0.009 (0.008) | 0.011 (0.016) |

Table S10: Performance of Survival Neural Networks (SNN) tuned for IBS at 5 years or C-index (61% censoring-2). The standard deviation based on 1000 datasets is provided in parentheses.

## For average censoring 20%

| **N_2_ = 1000** | **PLANN original**  **IBS** | **PLANN original**  **C-index** | **PLANN extended**  **IBS** | **PLANN extended**  **C-index** |
| --- | --- | --- | --- | --- |
| Brier 2 years (sd) | 0.143 (0.011) | 0.144 (0.011) | 0.143 (0.011) | 0.143 (0.010) |
| Brier 5 years (sd) | 0.227 (0.008) | 0.230 (0.009) | 0.227 (0.007) | 0.227 (0.006) |
| IBS 5 years (sd) | 0.123 (0.006) | 0.124 (0.007) | 0.123 (0.006) | 0.123 (0.006) |
| C-index (sd) | 0.617 (0.016) | 0.609 (0.021) | 0.617 (0.016) | 0.618 (0.016) |
| Miscalibration  2 years (sd) | 0.002 (0.002) | 0.002 (0.002) | 0.002 (0.002) | 0.003 (0.002) |
| Miscalibration  5 years (sd) | 0.004 (0.003) | 0.005 (0.003) | 0.004 (0.003) | 0.004 (0.003) |

Table S11: Performance of Survival Neural Networks (SNN) tuned for IBS at 5 years or C-index (20% censoring). The standard deviation based on 1000 datasets is provided in parentheses.

## For average censoring 40%

| **N_2_ = 1000** | **PLANN original**  **IBS** | **PLANN original**  **C-index** | **PLANN extended**  **IBS** | **PLANN extended**  **C-index** |
| --- | --- | --- | --- | --- |
| Brier 2 years (sd) | 0.144 (0.012) | 0.145 (0.012) | 0.144 (0.012) | 0.144 (0.012) |
| Brier 5 years (sd) | 0.228 (0.009) | 0.230 (0.009) | 0.231 (0.010) | 0.231 (0.011) |
| IBS 5 years (sd) | 0.123 (0.007) | 0.124 (0.007) | 0.124 (0.007) | 0.124 (0.007) |
| C-index (sd) | 0.622 (0.020) | 0.620 (0.021) | 0.623 (0.021) | 0.624 (0.019) |
| Miscalibration  2 years (sd) | 0.003 (0.003) | 0.003 (0.003) | 0.003 (0.003) | 0.003 (0.003) |
| Miscalibration  5 years (sd) | 0.005 (0.004) | 0.006 (0.004) | 0.008 (0.007) | 0.007 (0.007) |

Table S12: Performance of Survival Neural Networks (SNN) tuned for IBS at 5 years or C-index (40% censoring). The standard deviation based on 1000 datasets is provided in parentheses.

## For average censoring 80%

| **N_2_ = 1000** | **PLANN original**  **IBS** | **PLANN original**  **C-index** | **PLANN extended**  **IBS** | **PLANN extended**  **C-index** |
| --- | --- | --- | --- | --- |
| Brier 2 years (sd) | 0.151 (0.019) | 0.155 (0.020) | 0.146 (0.017) | 0.148 (0.017) |
| Brier 5 years (sd) | 0.244 (0.021) | 0.248 (0.023) | 0.236 (0.020) | 0.244 (0.017) |
| IBS 5 years (sd) | 0.131 (0.012) | 0.134 (0.013) | 0.126 (0.011) | 0.129 (0.011) |
| C-index (sd) | 0.646 (0.033) | 0.638 (0.034) | 0.646 (0.035) | 0.596 (0.069) |
| Miscalibration  2 years (sd) | 0.010 (0.007) | 0.014 (0.009) | 0.006 (0.005) | 0.006 (0.009) |
| Miscalibration  5 years (sd) | 0.023 (0.014) | 0.026 (0.015) | 0.016 (0.014) | 0.019 (0.015) |

Table S13: Performance of Survival Neural Networks (SNN) tuned for IBS at 5 years or C-index (80% censoring). The standard deviation based on 1000 datasets is provided in parentheses.

# Comparison of predictive performance for the methods: Brier score, C-index, IBS, and miscalibration

## For average censoring 61% -2


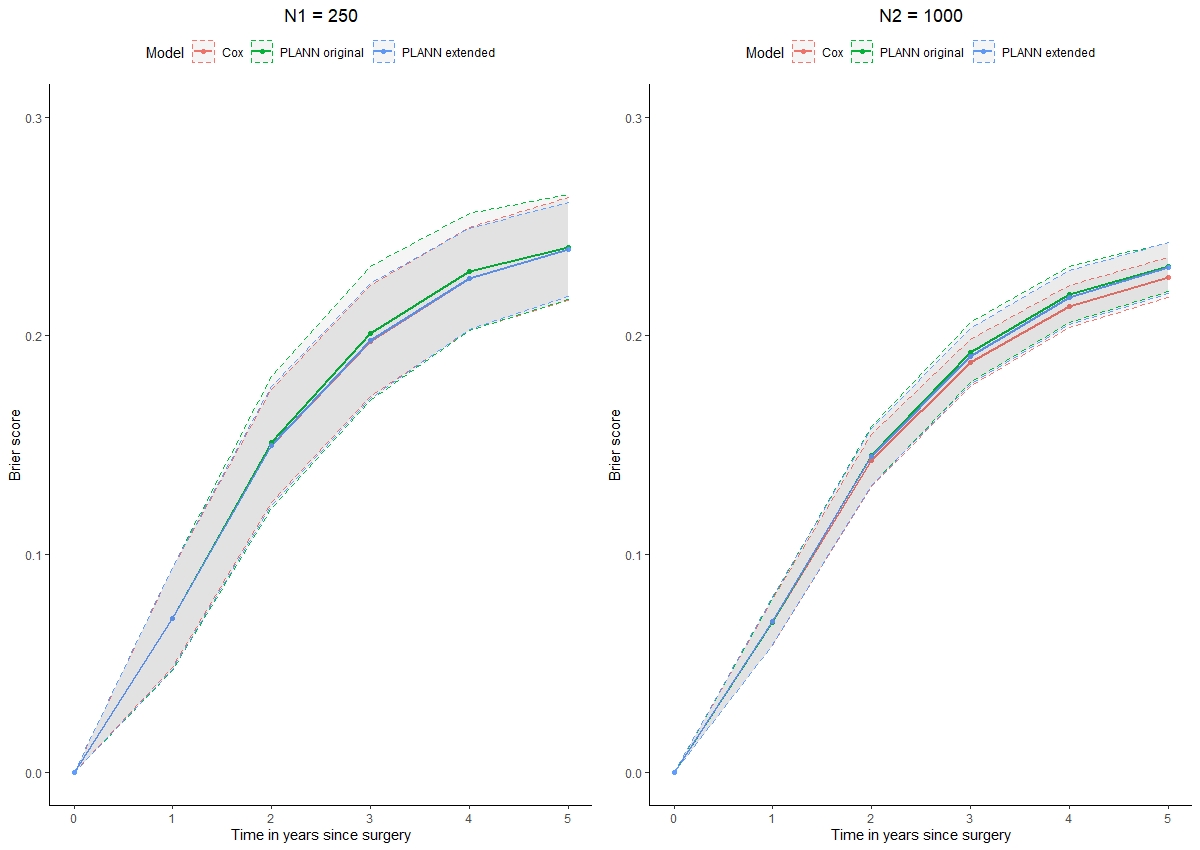


Figure S6: Brier score of the 3 methods (Cox, PLANN original, PLANN extended) ± one standard deviation for 61% censoring-2. Left panel: 250 patients, right panel: 1000 patients.


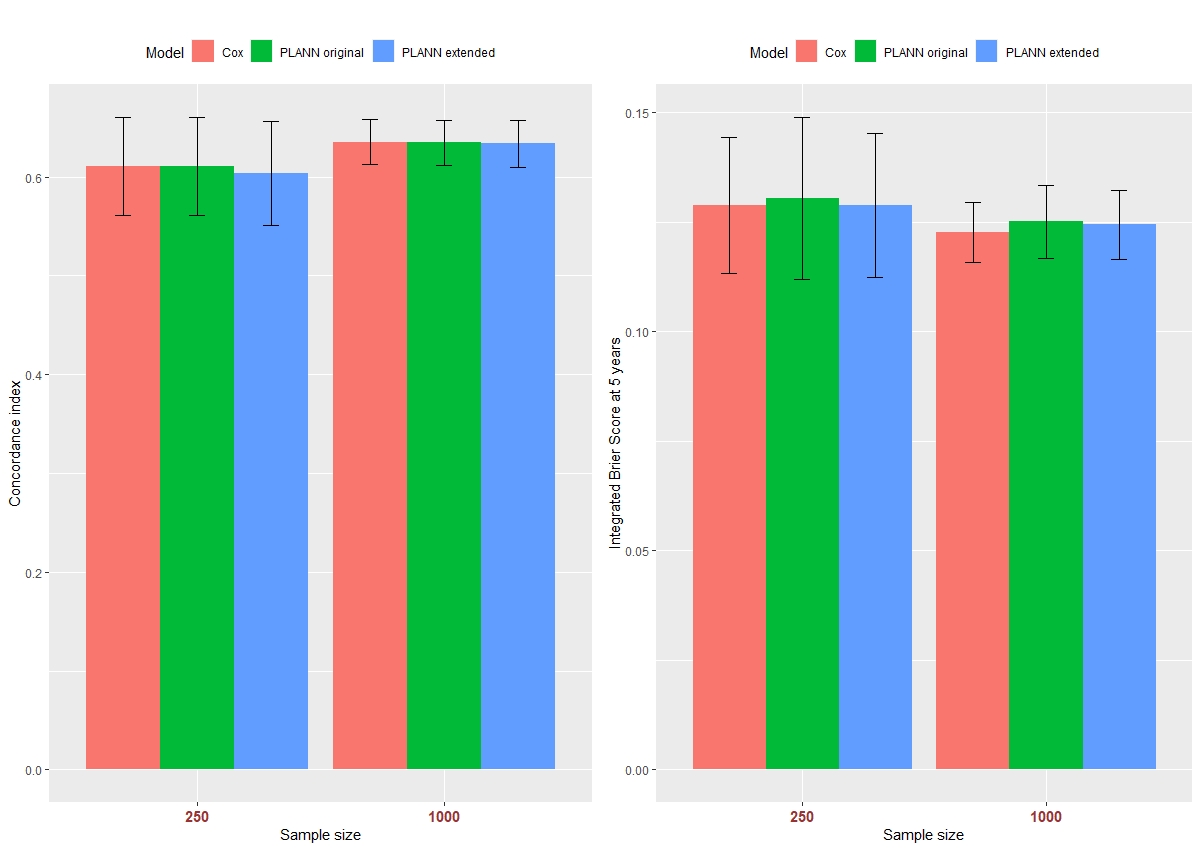


Figure S7: Predictive performance of the methods ± one standard deviation per sample size for 61% censoring-2. Left panel: C-index, right panel: IBS at 5 years.


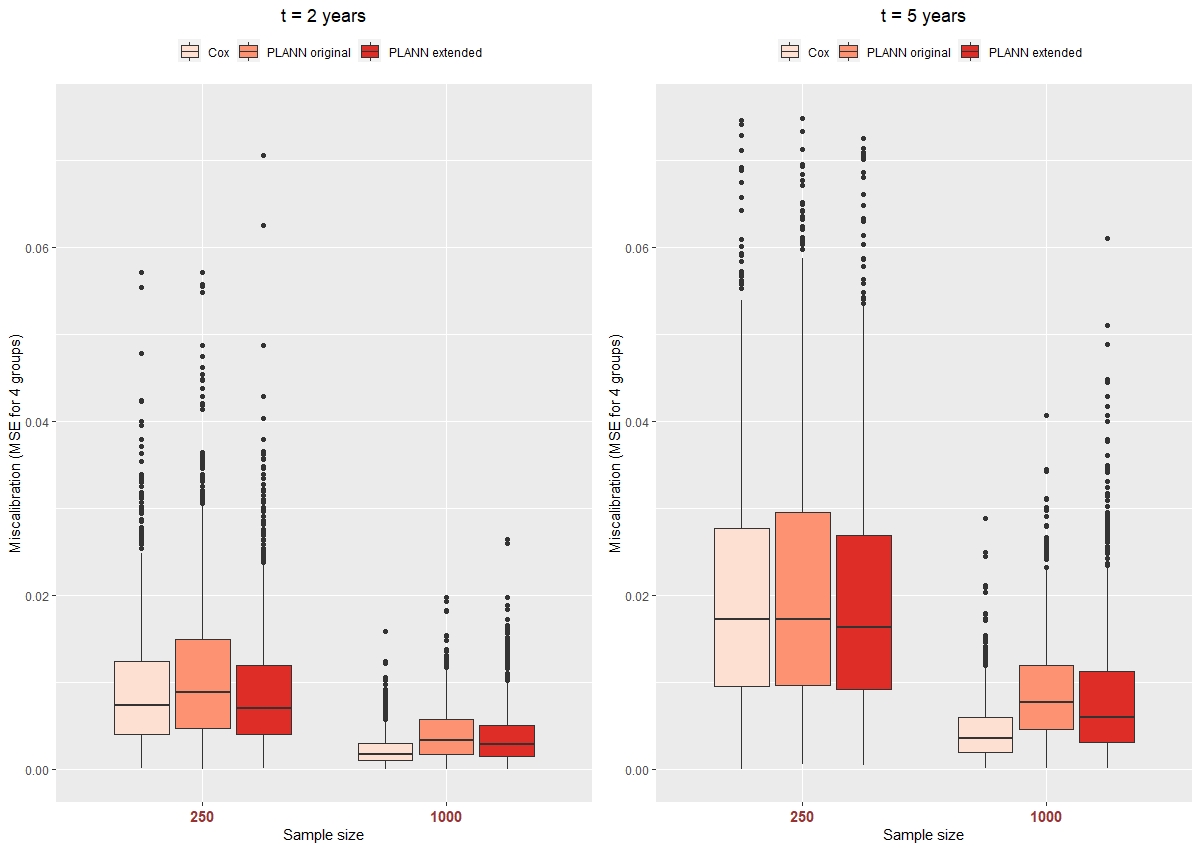


Figure S8: Miscalibration of the methods per sample size for 61% censoring-2. Left panel: at 2 years, right panel: at 5 years.

## For average censoring 20%


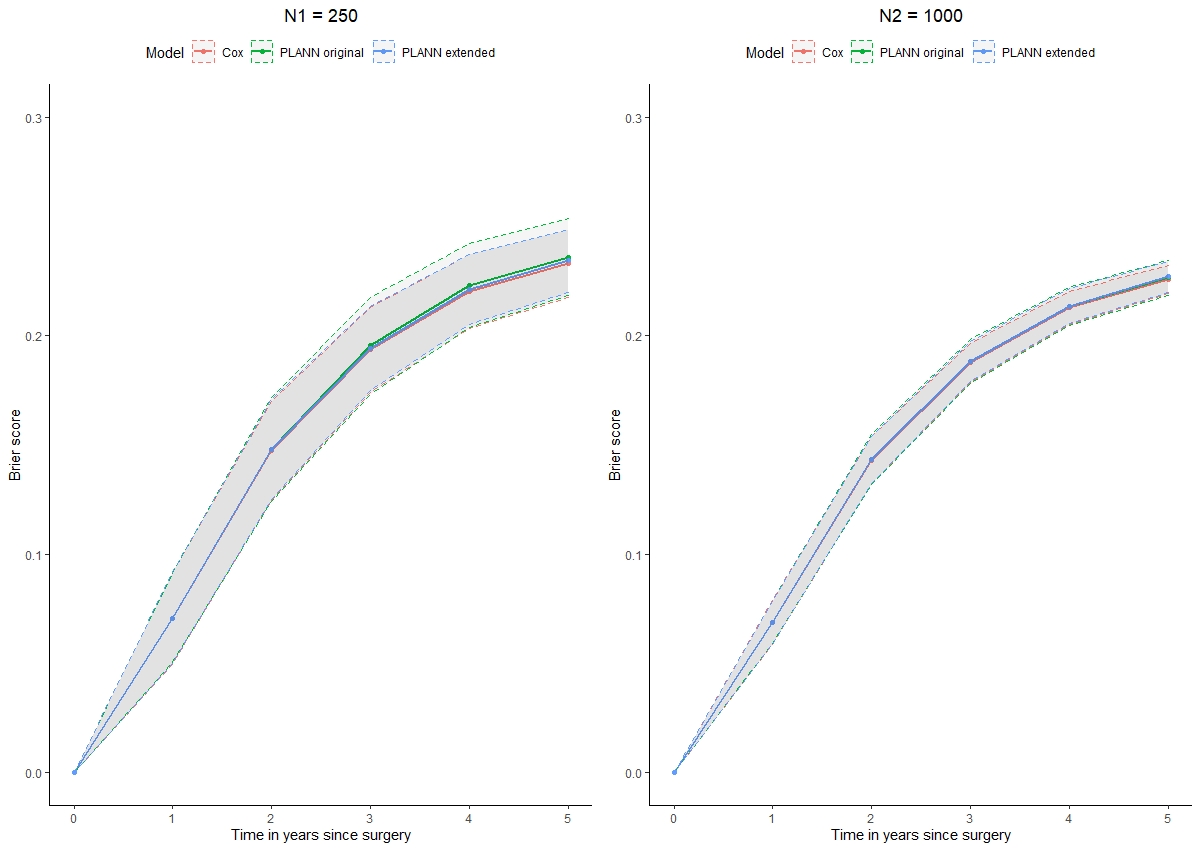


Figure S9: Brier score of the 3 methods (Cox, PLANN original, PLANN extended) ± one standard deviation for 20% censoring. Left panel: 250 patients, right panel: 1000 patients.


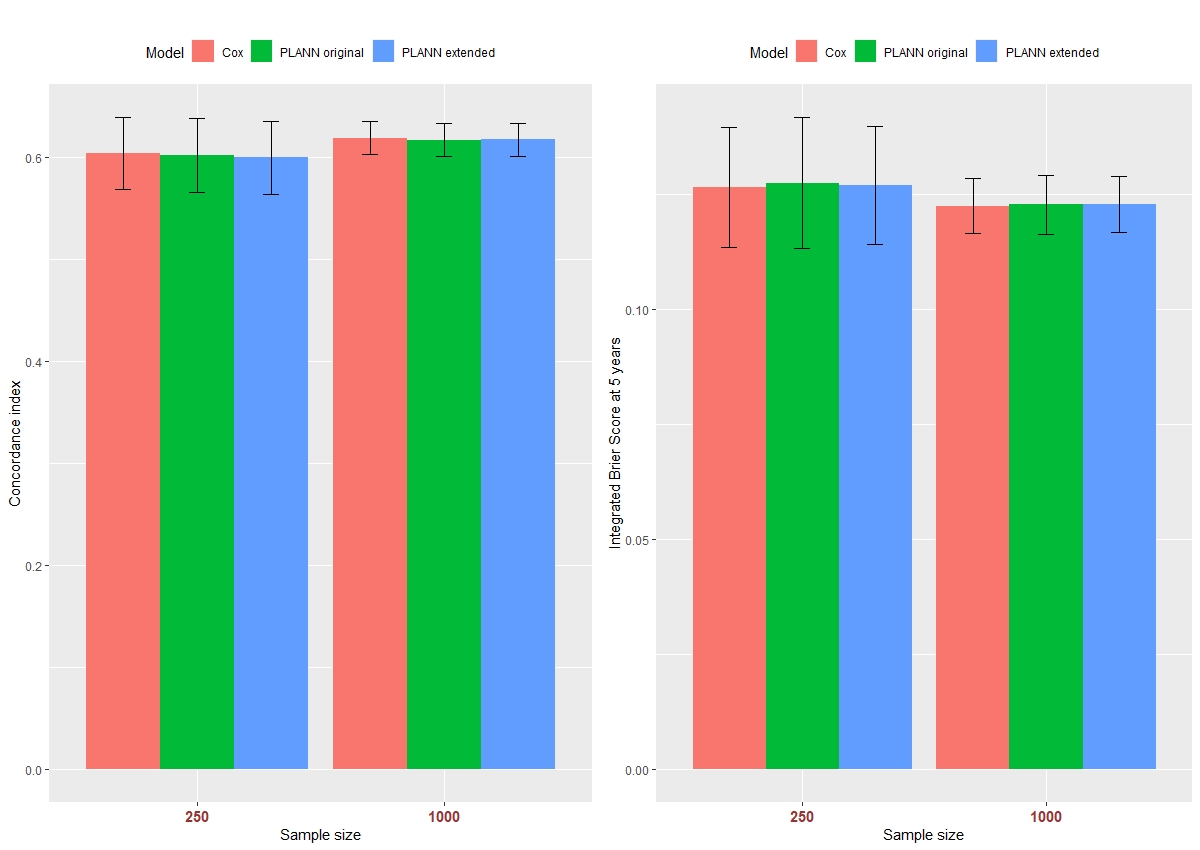


Figure S10: Predictive performance of the methods ± one standard deviation per sample size for 20% censoring. Left panel: C-index, right panel: IBS at 5 years.


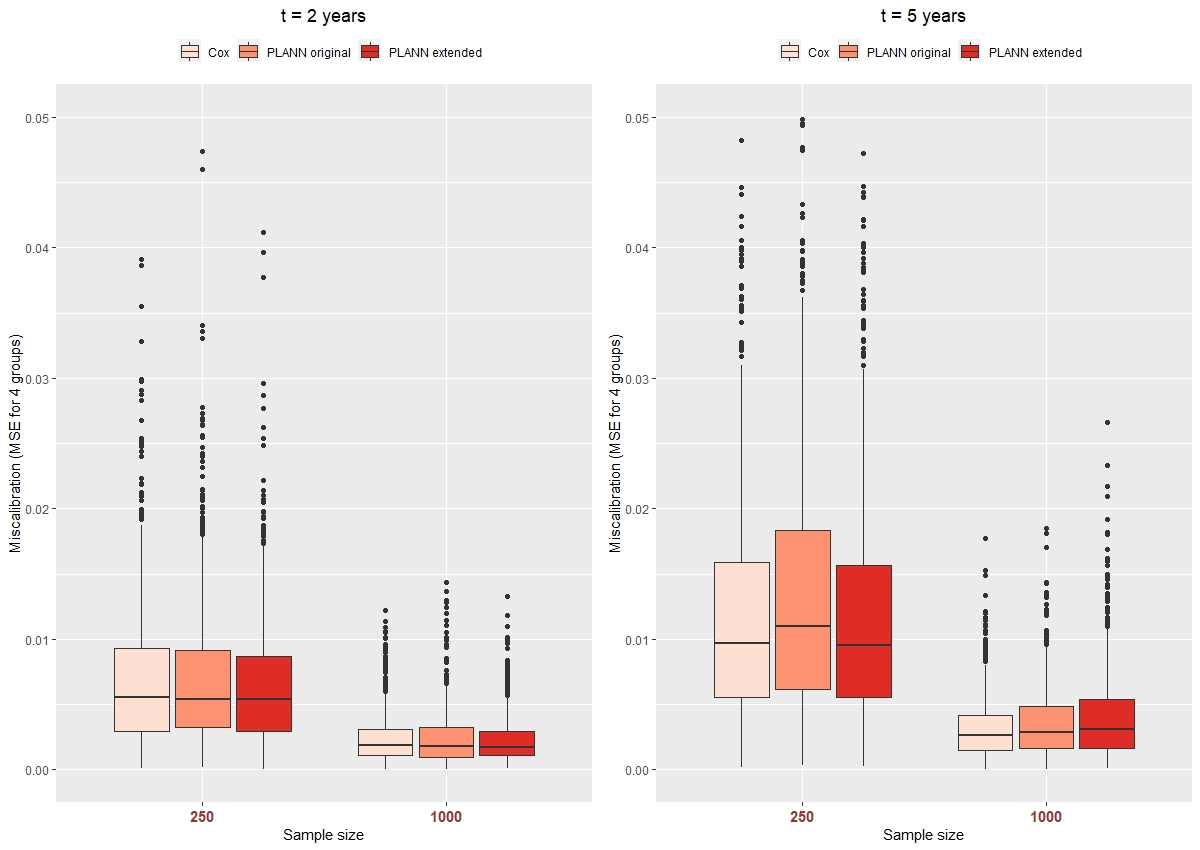


Figure S11: Miscalibration of the methods per sample size for 20% censoring. Left panel: at 2 years, right panel: at 5 years.

## For average censoring 40%


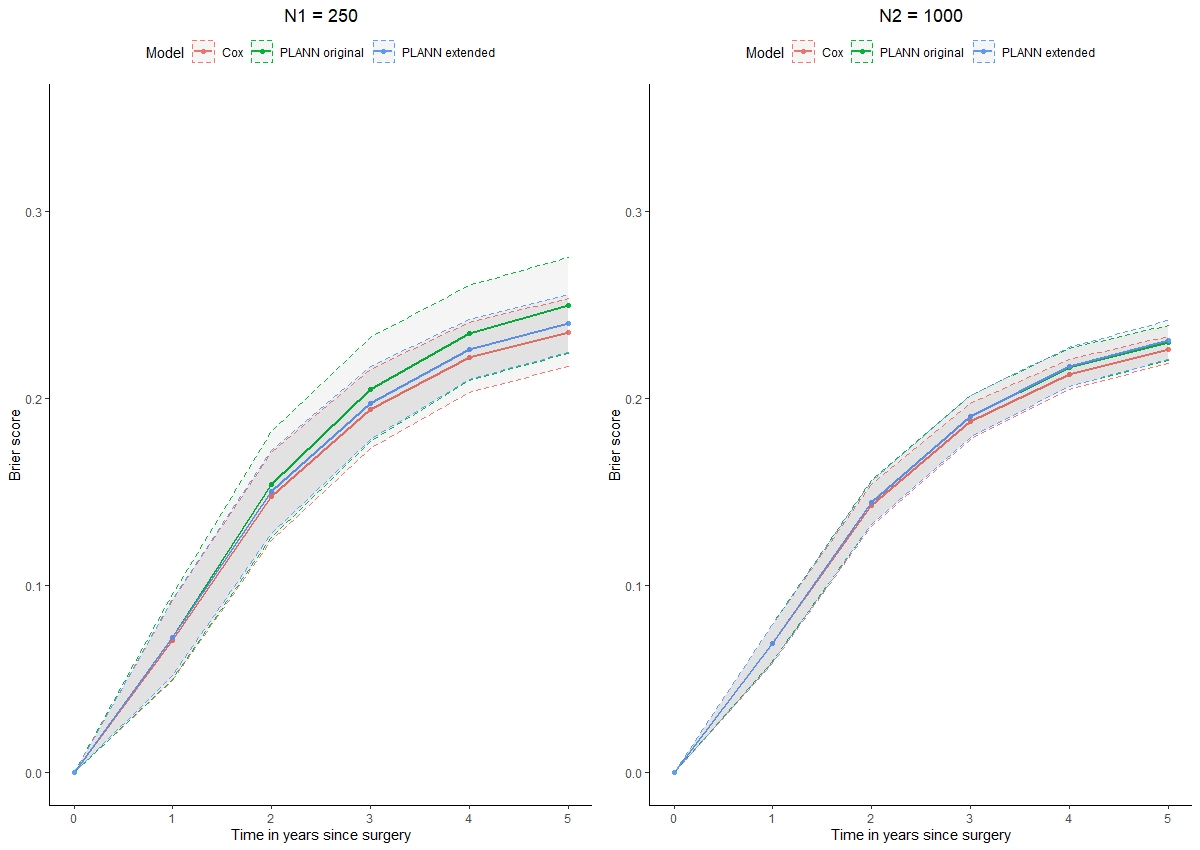


Figure S12: Brier score of the 3 methods (Cox, PLANN original, PLANN extended) ± one standard deviation for 40% censoring. Left panel: 250 patients, right panel: 1000 patients.


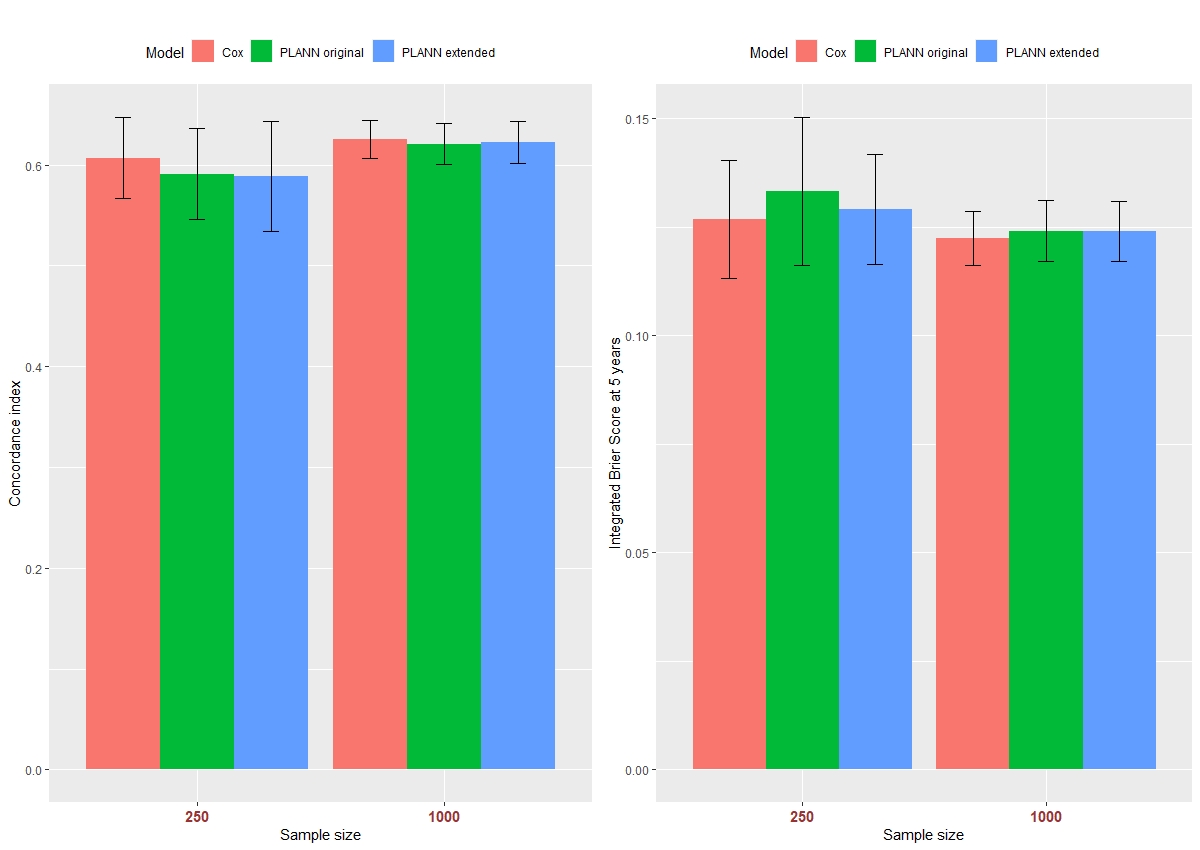


Figure S13: Predictive performance of the methods ± one standard deviation per sample size for 40% censoring. Left panel: C-index, right panel: IBS at 5 years.


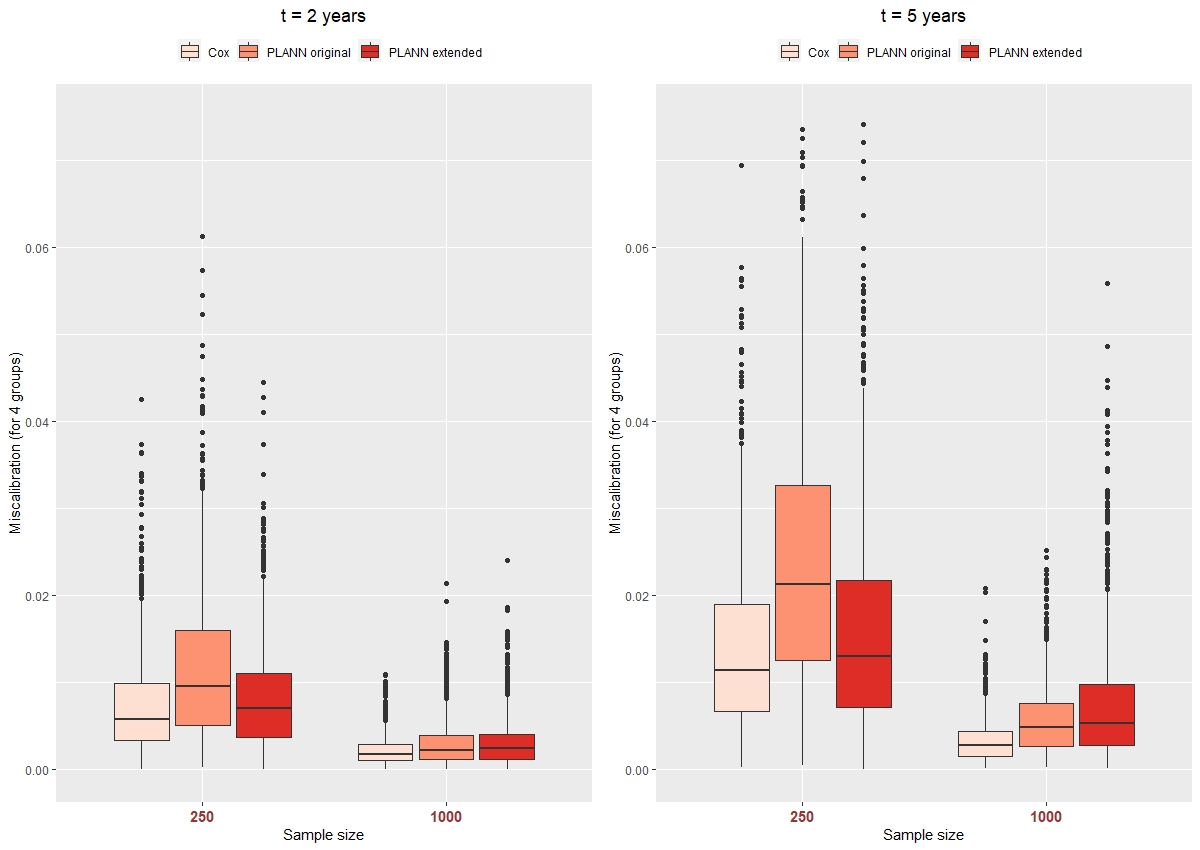


Figure S14: Miscalibration of the methods per sample size for 40% censoring. Left panel: at 2 years, right panel: at 5 years.

## For average censoring 80%


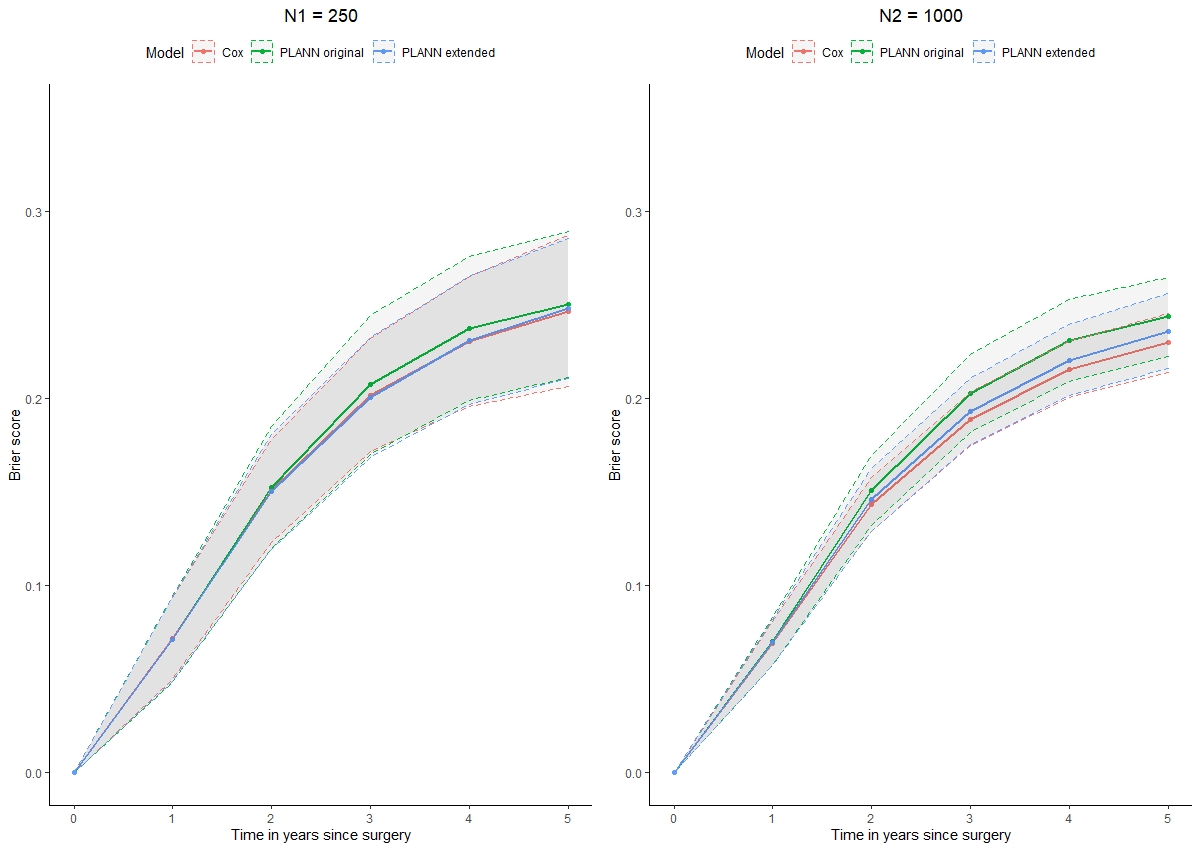


Figure S15: Brier score of the 3 methods (Cox, PLANN original, PLANN extended) ± one standard deviation for 80% censoring. Left panel: 250 patients, right panel: 1000 patients.


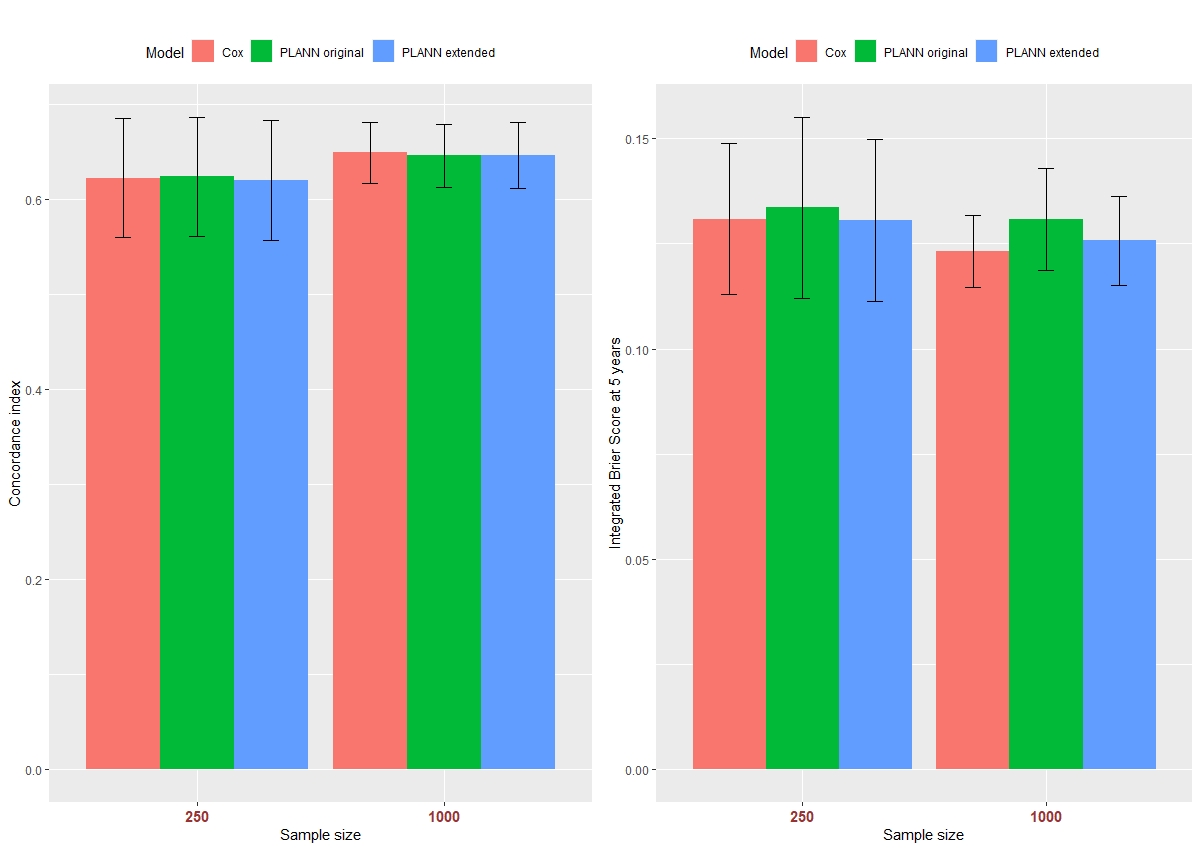


Figure S16: Predictive performance of the methods ± one standard deviation per sample size for 80% censoring. Left panel: C-index, right panel: IBS at 5 years.


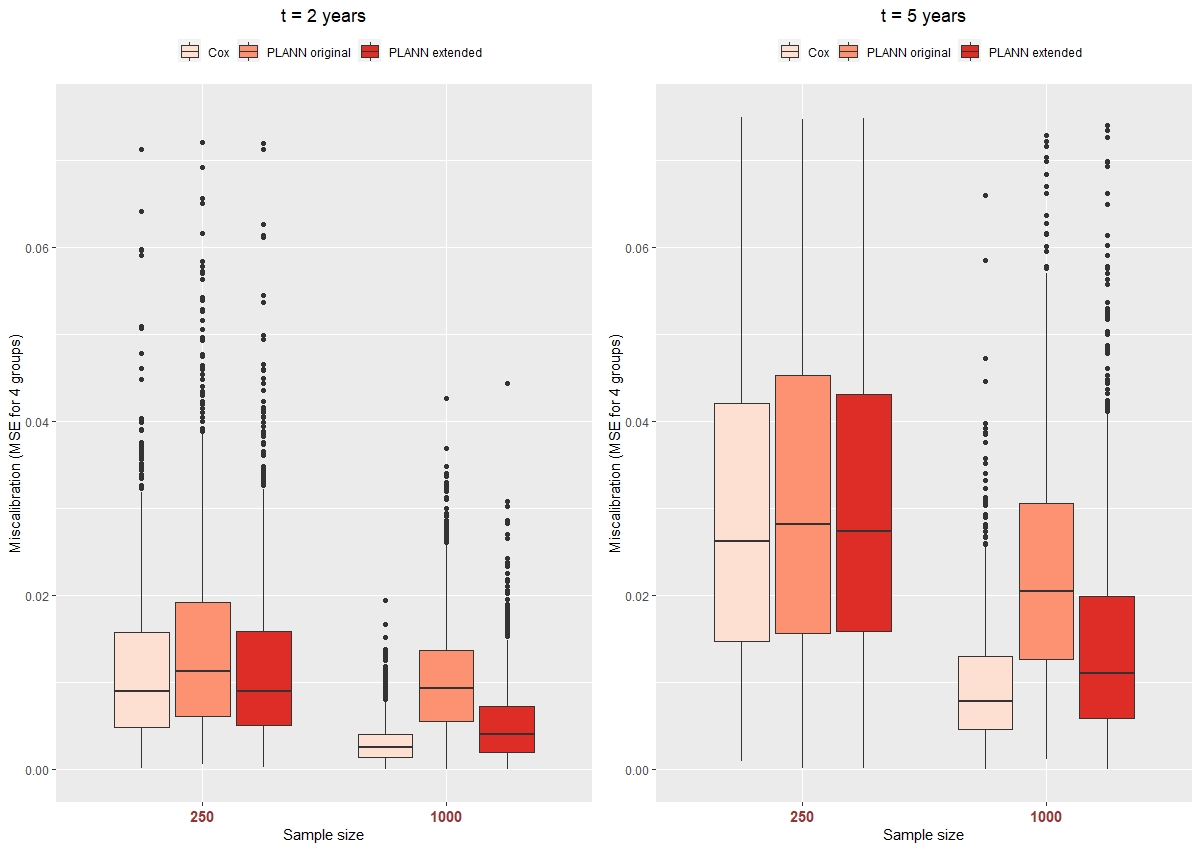


Figure S17: Miscalibration of the methods per sample size for 80% censoring. Left panel: at 2 years, right panel: at 5 years.

# Impact of adverse scenarios in predictive ability of the methods

To investigate the robustness of the survival neural networks, we defined the following 2 scenarios on the training part of the simulated data: i) Removing patients censored before the second year, or ii) curtailing patients’ survival at 5 years (administrative censoring). Results are presented below.

## For average censoring 61% -1

| **N_1_ = 250** | Min. | 1^st^ Qu. | Median | Mean | 3^rd^ Qu. | Max. |
| --- | --- | --- | --- | --- | --- | --- |
| Removing patients censored before 2^nd^ year | 4 | 10 | 12 | 12.43 | 15 | 24 |
| Curtailing survival at 5 years | 20 | 31 | 34 | 34.1 | 37 | 52 |
| **N_2_ = 1000** |  |  |  |  |  |  |
| Removing patients censored before 2^nd^ year | 28.00 | 45 | 50 | 49.74 | 54 | 71 |
| Curtailing survival at 5 years | 103 | 129 | 136 | 135.8 | 142 | 174 |

Table S14: Number of patients removed or curtailed per training dataset for 61% censoring-1.


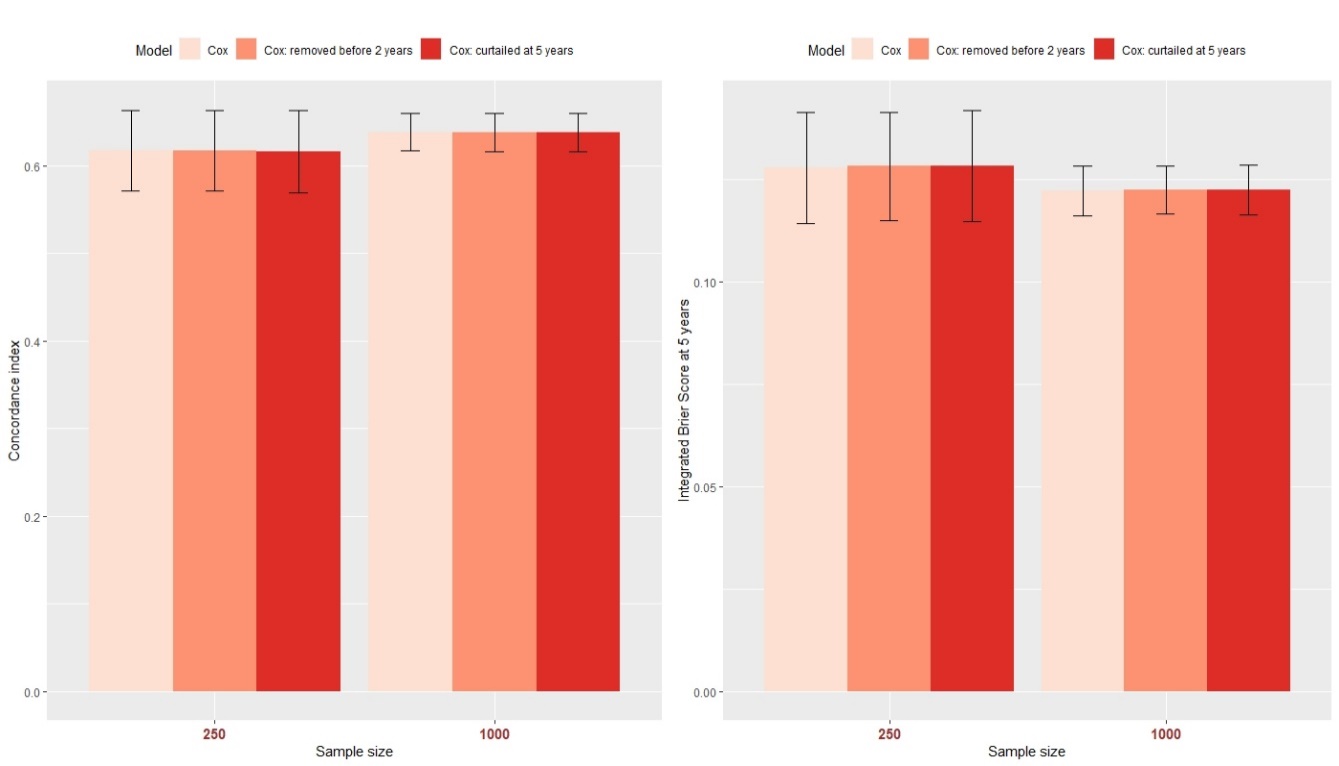


Figure S18: Predictive performance of Cox model ± one standard deviation per sample size for 61% censoring-1. Darker red palette colours correspond to the 2 adverse scenarios. Left panel: C-index, right panel: IBS at 5 years.

## For average censoring 61% -2

| **N_1_ = 250** | Min. | 1^st^ Qu. | Median | Mean | 3^rd^ Qu. | Max. |
| --- | --- | --- | --- | --- | --- | --- |
| Removing patients censored before 2^nd^ year | 24 | 35 | 39 | 38.7 | 42 | 59 |
| Curtailing survival at 5 years | 18 | 30 | 33 | 33.03 | 36 | 51 |
| **N_2_ = 1000** |  |  |  |  |  |  |
| Removing patients censored before 2^nd^ year | 119 | 148 | 155 | 155 | 162 | 190 |
| Curtailing survival at 5 years | 96 | 125 | 132 | 131.5 | 138 | 167 |

Table S15: Number of patients removed or curtailed per training dataset for 61% censoring-2.


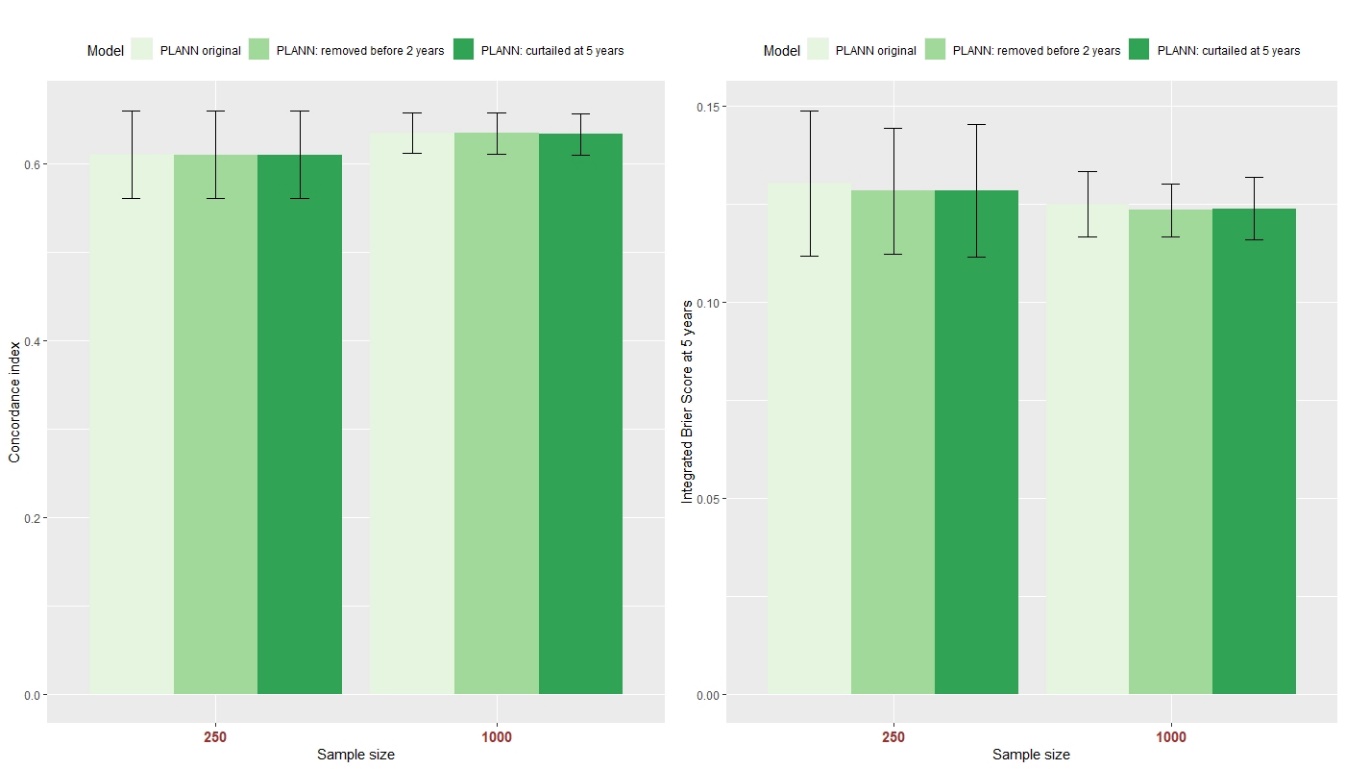


Figure S19: Predictive performance of PLANN original ± one standard deviation per sample size for 61% censoring-2. Darker green palette colours correspond to the 2 adverse scenarios. Left panel: C-index, right panel: IBS at 5 years.


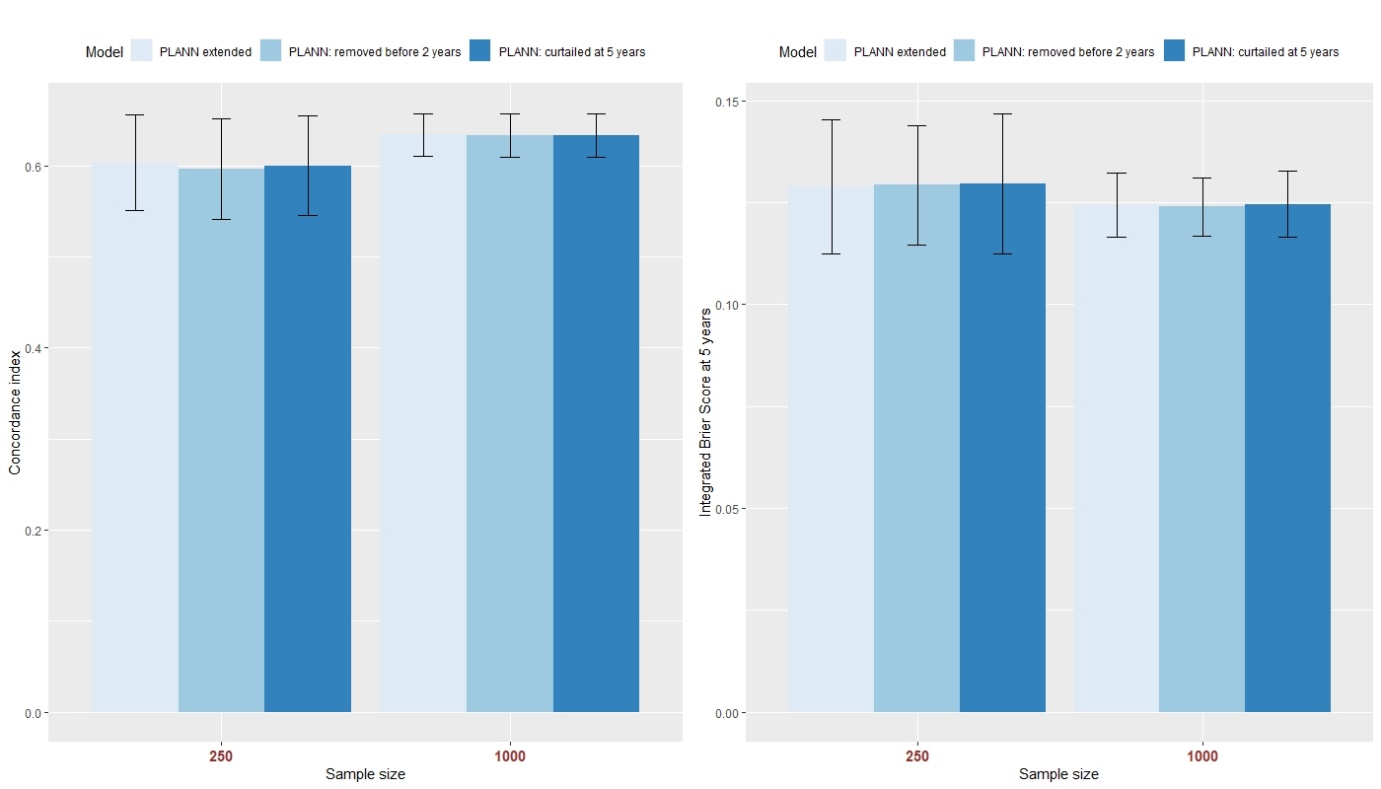


Figure S20: Predictive performance of PLANN extended ± one standard deviation per sample size for 61% censoring-2. Darker blue palette colours correspond to the 2 adverse scenarios. Left panel: C-index, right panel: IBS at 5 years.


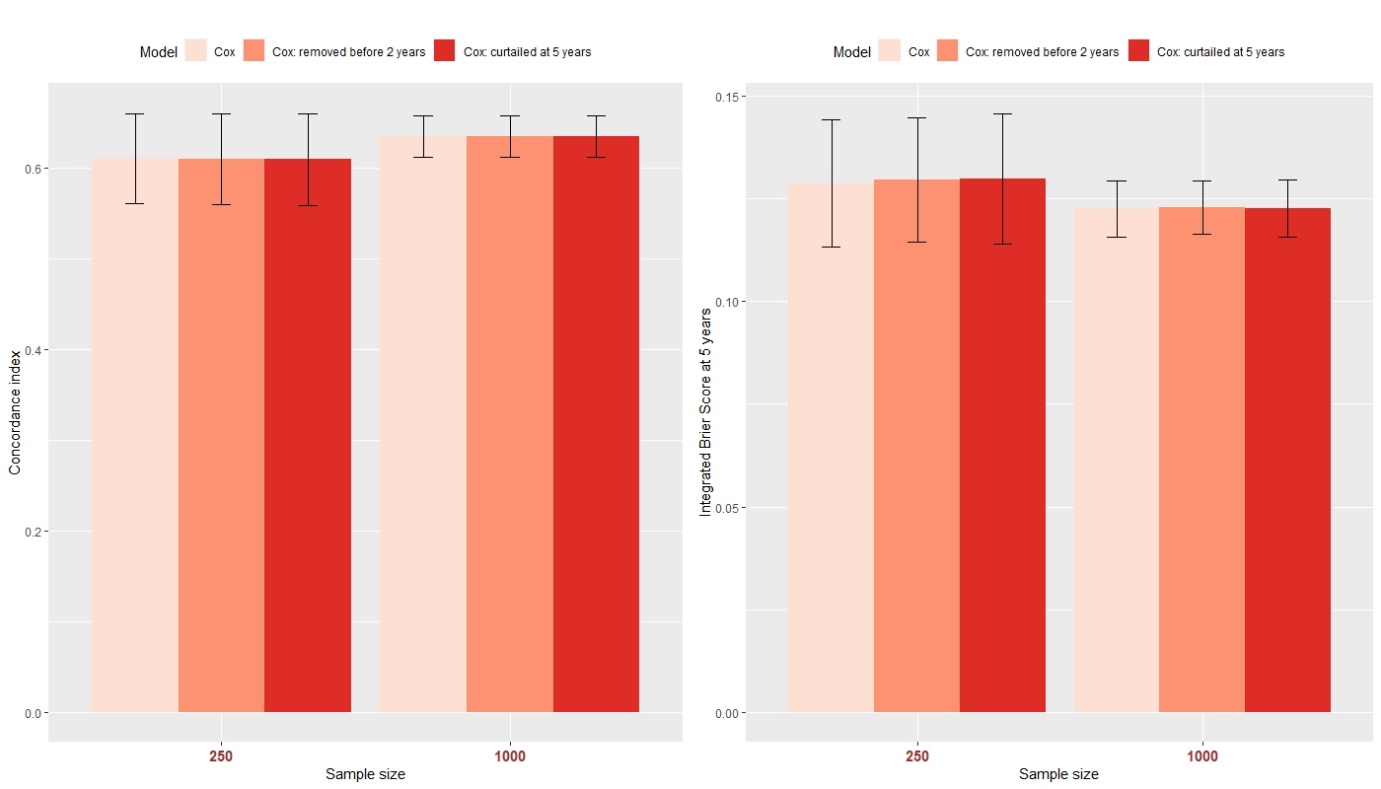


Figure S21: Predictive performance of Cox model ± one standard deviation per sample size for 61% censoring-2. Darker red palette colours correspond to the 2 adverse scenarios. Left panel: C-index, right panel: IBS at 5 years.

## For average censoring 20%

| **N_1_ = 250** | Min. | 1^st^ Qu. | Median | Mean | 3^rd^ Qu. | Max. |
| --- | --- | --- | --- | --- | --- | --- |
| Removing patients censored before 2^nd^ year | 1 | 5 | 7 | 7.37 | 9 | 16 |
| Curtailing survival at 5 years | 48 | 60 | 64 | 64 | 68 | 81 |
| **N_2_ = 1000** |  |  |  |  |  |  |
| Removing patients censored before 2^nd^ year | 15 | 26 | 29 | 29.49 | 33 | 51 |
| Curtailing survival at 5 years | 220 | 248 | 255 | 255.6 | 263 | 295 |

Table S16: Number of patients removed or curtailed per training dataset for 20% censoring.


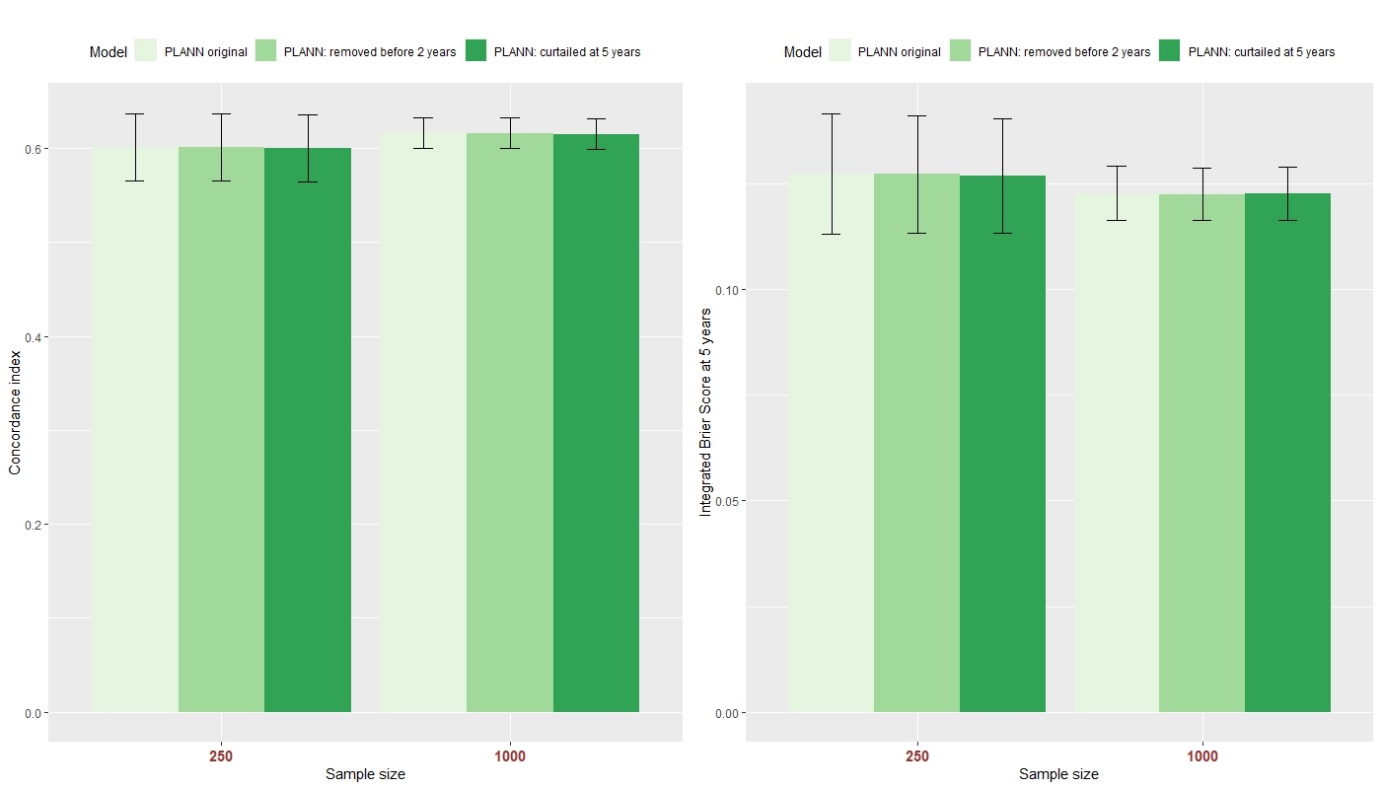


Figure S22: Predictive performance of PLANN original ± one standard deviation per sample size for 20% censoring. Darker green palette colours correspond to the 2 adverse scenarios. Left panel: C-index, right panel: IBS at 5 years.


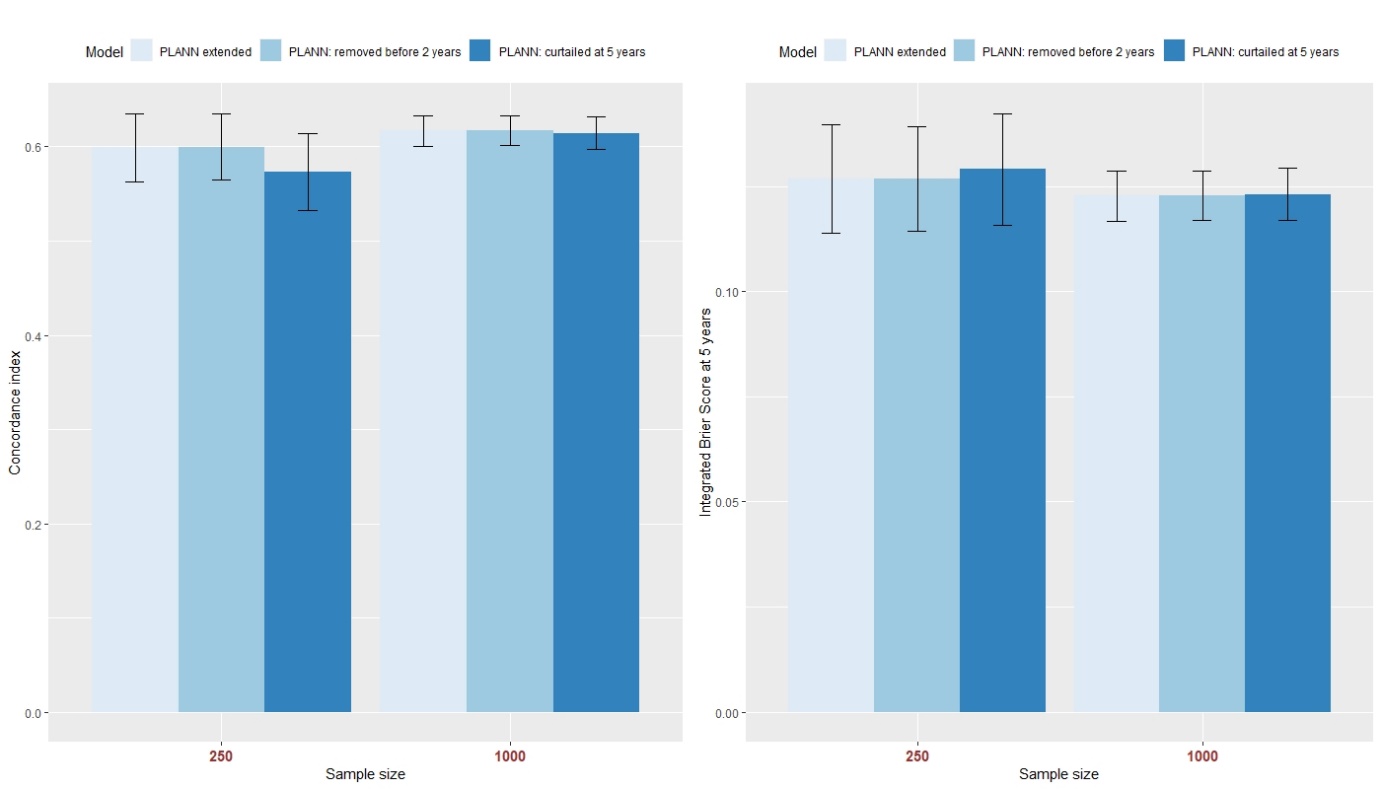


Figure S23: Predictive performance of PLANN extended ± one standard deviation per sample size for 20% censoring. Darker blue palette colours correspond to the 2 adverse scenarios. Left panel: C-index, right panel: IBS at 5 years.


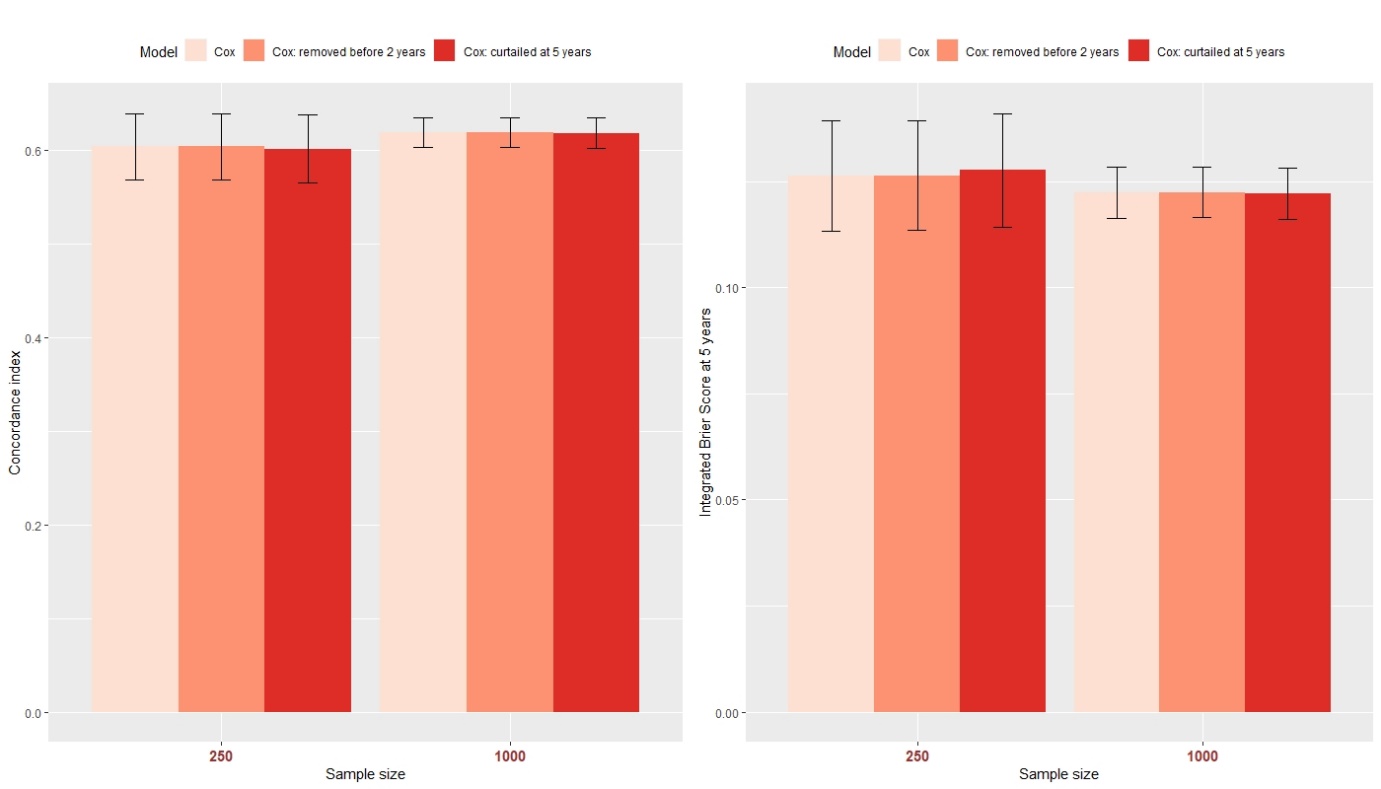


Figure S24: Predictive performance of Cox model ± one standard deviation per sample size for 20% censoring. Darker red palette colours correspond to the 2 adverse scenarios. Left panel: C-index, right panel: IBS at 5 years.

## For average censoring 40%

| **N_1_ = 250** | Min. | 1^st^ Qu. | Median | Mean | 3^rd^ Qu. | Max. |
| --- | --- | --- | --- | --- | --- | --- |
| Removing patients censored before 2^nd^ year | 7 | 16 | 19 | 18.72 | 21 | 31 |
| Curtailing survival at 5 years | 34 | 48 | 51 | 51.62 | 55 | 67 |
| **N_2_ = 1000** |  |  |  |  |  |  |
| Removing patients censored before 2^nd^ year | 51 | 70 | 75 | 75.22 | 81 | 99 |
| Curtailing survival at 5 years | 169 | 198 | 205 | 205.4 | 212 | 247 |

Table S17: Number of patients removed or curtailed per training dataset for 40% censoring.


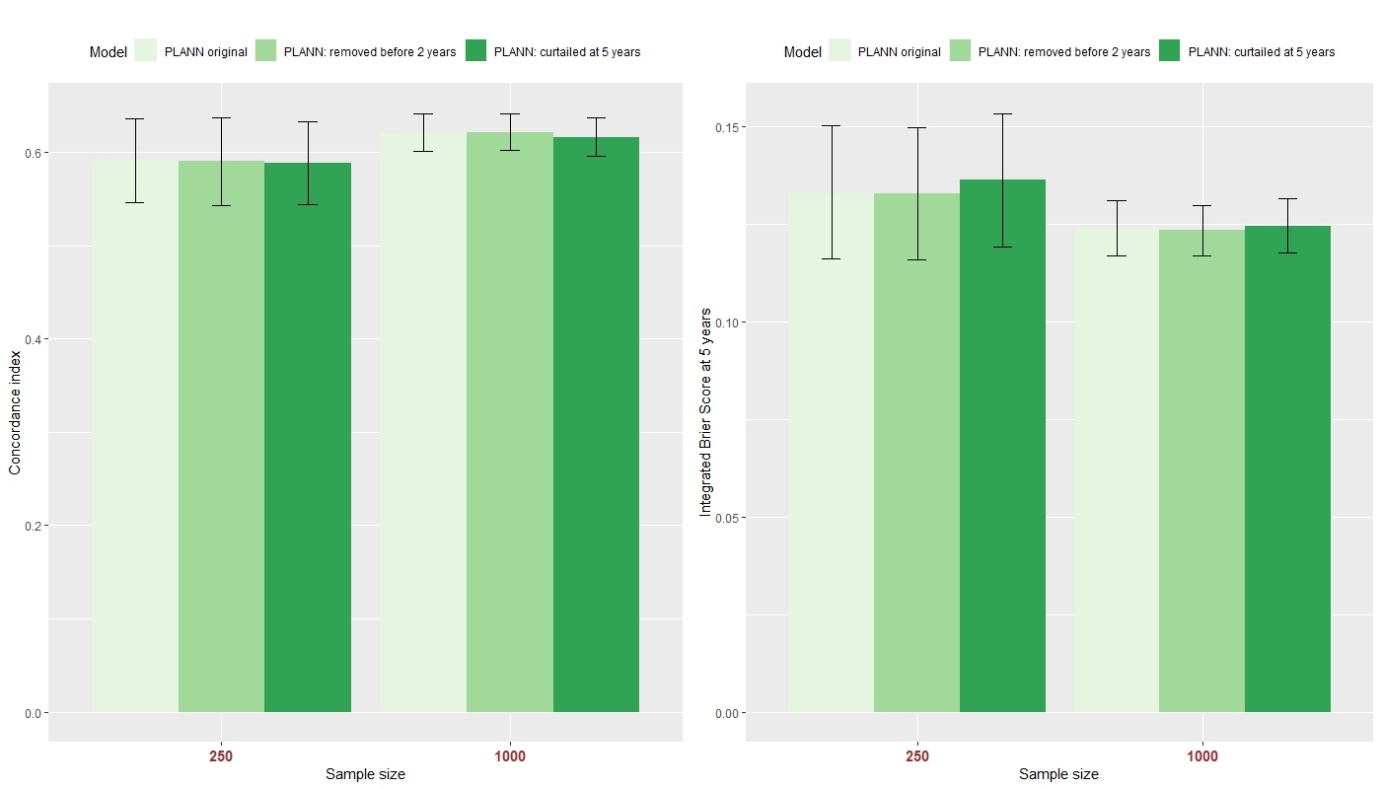


Figure S25: Predictive performance of PLANN original ± one standard deviation per sample size for 40% censoring. Darker green palette colours correspond to the 2 adverse scenarios. Left panel: C-index, right panel: IBS at 5 years.


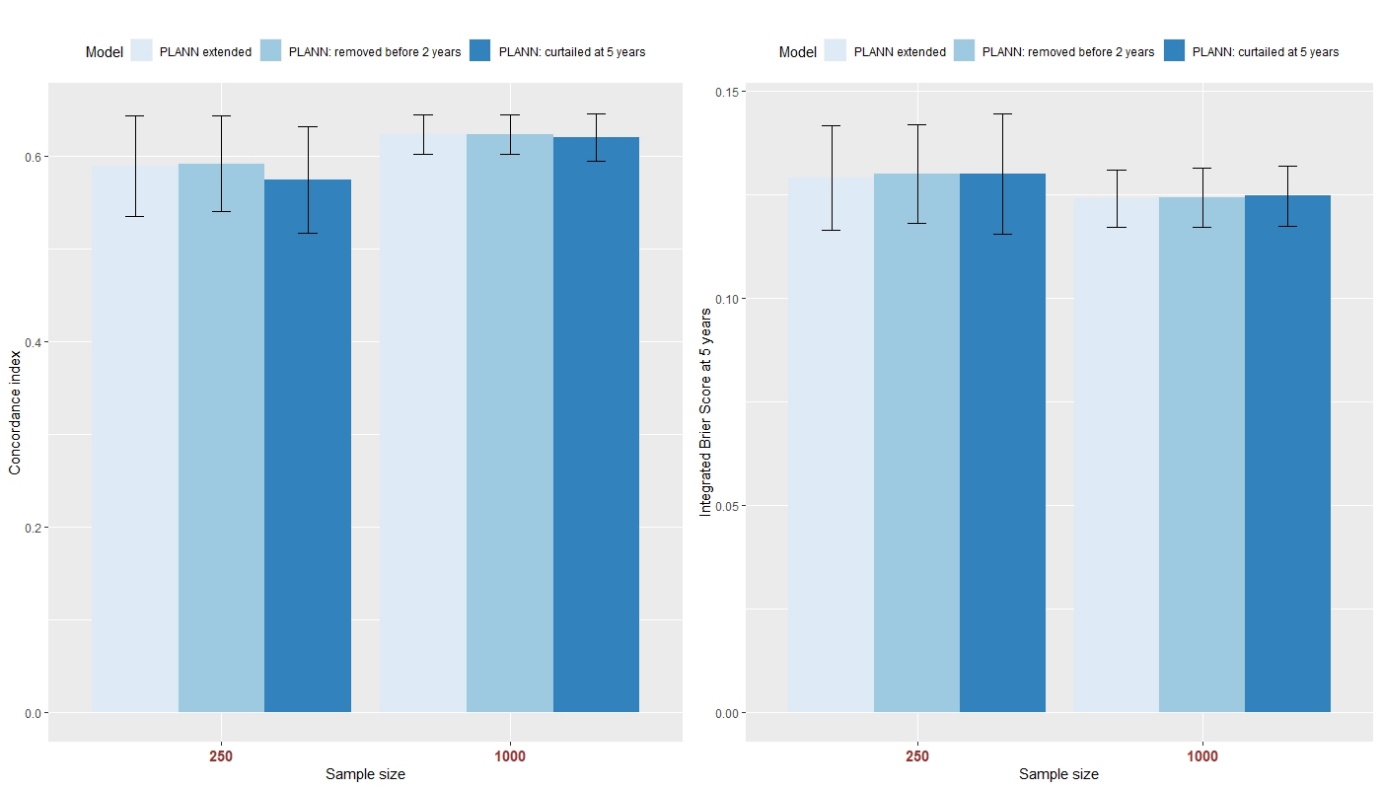


Figure S26: Predictive performance of PLANN extended ± one standard deviation per sample size for 40% censoring. Darker blue palette colours correspond to the 2 adverse scenarios. Left panel: C-index, right panel: IBS at 5 years.


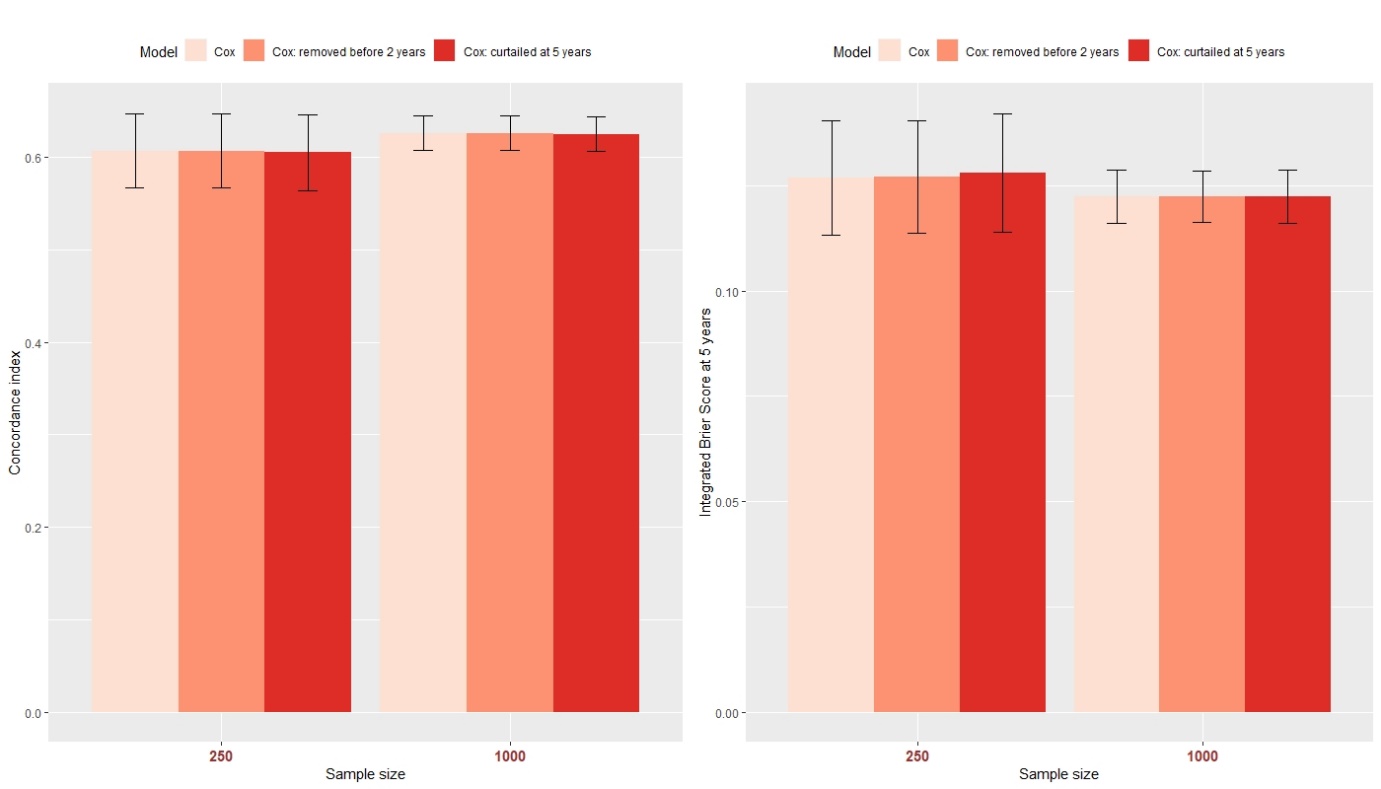


Figure S27: Predictive performance of Cox model ± one standard deviation per sample size for 40% censoring. Darker red palette colours correspond to the 2 adverse scenarios. Left panel: C-index, right panel: IBS at 5 years.

## For average censoring 80%

| **N_1_ = 250** | Min. | 1^st^ Qu. | Median | Mean | 3^rd^ Qu. | Max. |
| --- | --- | --- | --- | --- | --- | --- |
| Removing patients censored before 2^nd^ year | 65 | 79 | 83 | 82.88 | 87 | 101 |
| Curtailing survival at 5 years | 5 | 13 | 15 | 15.37 | 18 | 29 |
| **N_2_ = 1000** |  |  |  |  |  |  |
| Removing patients censored before 2^nd^ year | 163 | 191 | 199 | 198.3 | 206 | 236 |
| Curtailing survival at 5 years | 31 | 47 | 51 | 51.28 | 56 | 73 |

Table S18: Number of patients removed or curtailed per training dataset for 80% censoring.


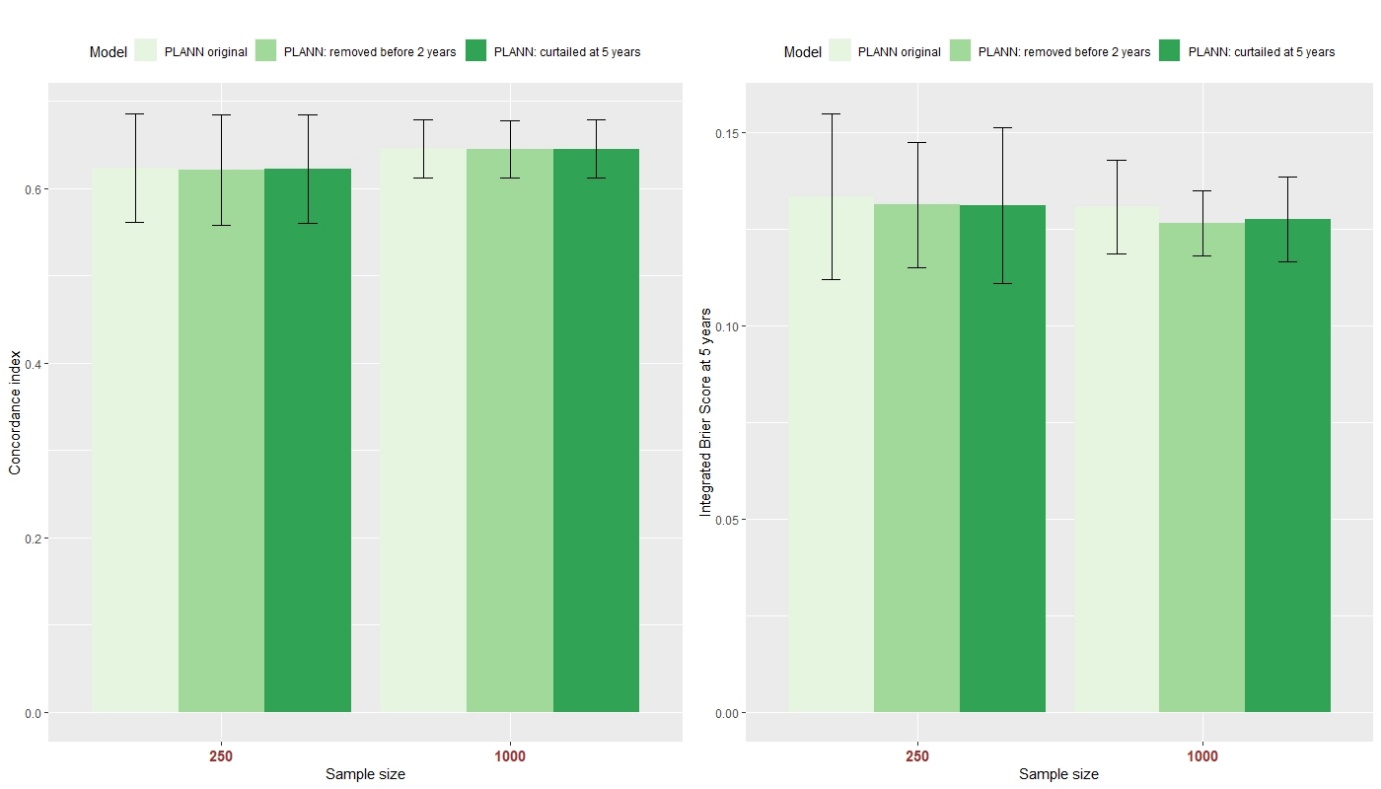


Figure S28: Predictive performance of PLANN original ± one standard deviation per sample size for 80% censoring. Darker green palette colours correspond to the 2 adverse scenarios. Left panel: C-index, right panel: IBS at 5 years.


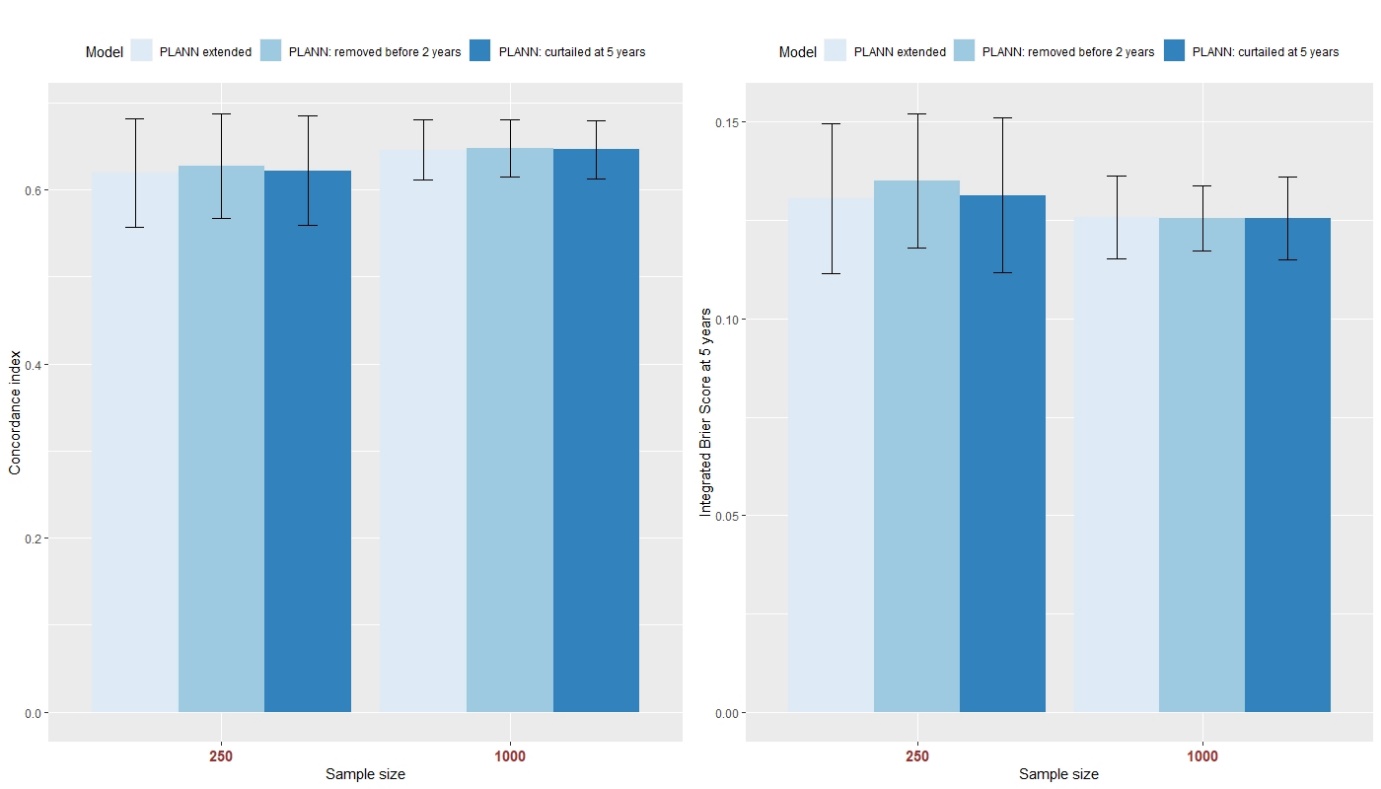


Figure S29: Predictive performance of PLANN extended ± one standard deviation per sample size for 80% censoring. Darker blue palette colours correspond to the 2 adverse scenarios. Left panel: C-index, right panel: IBS at 5 years.


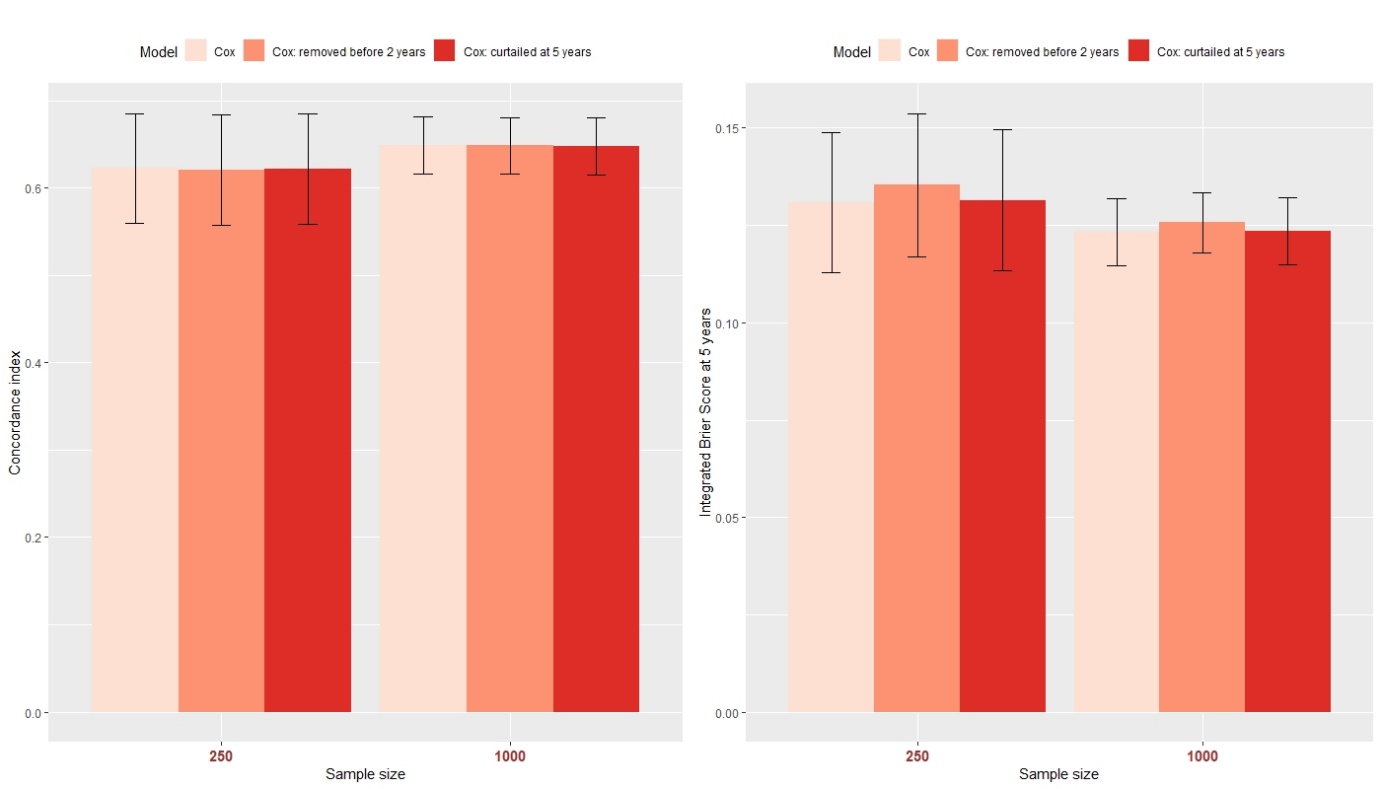


Figure S30: Predictive performance of Cox model ± one standard deviation per sample size for 80% censoring. Darker red palette colours correspond to the 2 adverse scenarios. Left panel: C-index, right panel: IBS at 5 years.

# Effect of interval length for SNN: 61% censoring-1

The networks below were employed on B = 1000 simulated datasets with the optimal parameters chosen based on IBS.

## PLANN original

| **N_1_ = 250** | **3-monthly** | **6-monthly** | **yearly** |
| --- | --- | --- | --- |
| Brier 2 years (sd) | 0.148 (0.024) | 0.148 (0.024) | 0.148 (0.024) |
| Brier 5 years (sd) | 0.237 (0.021) | 0.237 (0.021) | 0.237 (0.021) |
| IBS (sd) | 0.128 (0.014) | 0.128 (0.014) | 0.128 (0.014) |
| C-index (sd) | 0.616 (0.046) | 0.616 (0.046) | 0.617 (0.046) |
| Miscalibration  2 years (sd) | 0.007 (0.006) | 0.007 (0.006) | 0.007 (0.006) |
| Miscalibration  5 years (sd) | 0.017 (0.012) | 0.017 (0.012) | 0.017 (0.013) |

Table S19: Predictive performance of PLANN original for 3-monthly, 6-monthly, or yearly time intervals and 250 synthetic patients per dataset with 61% censoring-1. The standard deviation based on 1000 datasets is provided in parentheses.

| **N_2_ = 1000** | **3-monthly** | **6-monthly** | **yearly** |
| --- | --- | --- | --- |
| Brier 2 years (sd) | 0.146 (0.012) | 0.146 (0.012) | 0.145 (0.012) |
| Brier 5 years (sd) | 0.229 (0.010) | 0.229 (0.010) | 0.229 (0.010) |
| IBS (sd) | 0.125 (0.007) | 0.124 (0.007) | 0.124 (0.007) |
| C-index (sd) | 0.633 (0.022) | 0.633 (0.022) | 0.633 (0.022) |
| Miscalibration  2 years (sd) | 0.004 (0.003) | 0.004 (0.003) | 0.003 (0.003) |
| Miscalibration  5 years (sd) | 0.006 (0.004) | 0.005 (0.004) | 0.006 (0.004) |

Table S20: Predictive performance of PLANN original for 3-monthly, 6-monthly, or yearly time intervals and 1000 synthetic patients per dataset with 61% censoring-1. The standard deviation based on 1000 datasets is provided in parentheses.

## PLANN extended

| **N_1_ = 250** | **3-monthly** | **6-monthly** | **yearly** |
| --- | --- | --- | --- |
| Brier 2 years (sd) | 0.152 (0.024) | 0.152 (0.024) | 0.149 (0.023) |
| Brier 5 years (sd) | 0.249 (0.028) | 0.249 (0.029) | 0.238 (0.021) |
| IBS (sd) | 0.132 (0.015) | 0.132 (0.016) | 0.128 (0.014) |
| C-index (sd) | 0.604 (0.050) | 0.616 (0.046) | 0.616 (0.045) |
| Miscalibration  2 years (sd) | 0.009 (0.008) | 0.011 (0.009) | 0.007 (0.006) |
| Miscalibration  5 years (sd) | 0.026 (0.020) | 0.028 (0.022) | 0.017 (0.013) |

Table S21: Predictive performance of PLANN extended for 3-monthly, 6-monthly, or yearly time intervals and 250 synthetic patients per dataset with 61% censoring-1. The standard deviation based on 1000 datasets is provided in parentheses.

| **N_2_ = 1000** | **3-monthly** | **6-monthly** | **yearly** |
| --- | --- | --- | --- |
| Brier 2 years (sd) | 0.146 (0.011) | 0.146 (0.012) | 0.144 (0.011) |
| Brier 5 years (sd) | 0.236 (0.012) | 0.236 (0.015) | 0.229 (0.011) |
| IBS (sd) | 0.126 (0.007) | 0.126 (0.008) | 0.123 (0.006) |
| C-index (sd) | 0.623 (0.025) | 0.634 (0.023) | 0.637 (0.021) |
| Miscalibration  2 years (sd) | 0.003 (0.003) | 0.004 (0.005) | 0.003 (0.002) |
| Miscalibration  5 years (sd) | 0.010 (0.008) | 0.012 (0.012) | 0.008 (0.006) |

Table S22: Predictive performance of PLANN extended for 3-monthly, 6-monthly, or yearly time intervals and 1000 synthetic patients per dataset with 61% censoring-1. The standard deviation based on 1000 datasets is provided in parentheses.

# Effect of different activation functions for PLANN extended: 61% censoring-1

The networks below were employed on B = 1000 simulated datasets with the optimal parameters chosen based on IBS (section 2). The hyperbolic tangent (tanh) activation function provided the best performance in terms of IBS on the training data as well as on the simulated datasets.

| **N_1_ = 250** | **sigmoid** | **relu** | **tanh** |
| --- | --- | --- | --- |
| Brier 2 years (sd) | 0.151 (0.023) | 0.151 (0.023) | 0.149 (0.023) |
| Brier 5 years (sd) | 0.245 (0.019) | 0.244 (0.019) | 0.238 (0.021) |
| IBS (sd) | 0.131 (0.014) | 0.130 (0.014) | 0.128 (0.014) |
| C-index (sd) | 0.575 (0.055) | 0.568 (0.066) | 0.616 (0.045) |
| Miscalibration  2 years (sd) | 0.008 (0.006) | 0.007 (0.006) | 0.007 (0.006) |
| Miscalibration  5 years (sd) | 0.019 (0.015) | 0.018 (0.014) | 0.017 (0.013) |

Table S23: Predictive performance of PLANN extended for different activations functions with 250 patients and 61% censoring-1. The standard deviation based on 1000 datasets is provided in parentheses.

| **N_2_ =1000** | **sigmoid** | **relu** | **tanh** |
| --- | --- | --- | --- |
| Brier 2 years (sd) | 0.144 (0.011) | 0.144 (0.011) | 0.144 (0.011) |
| Brier 5 years (sd) | 0.231 (0.011) | 0.231 (0.010) | 0.229 (0.011) |
| IBS (sd) | 0.124 (0.007) | 0.124 (0.007) | 0.123 (0.006) |
| C-index (sd) | 0.634 (0.023) | 0.626 (0.028) | 0.637 (0.021) |
| Miscalibration  2 years (sd) | 0.003 (0.002) | 0.003 (0.002) | 0.003 (0.002) |
| Miscalibration  5 years (sd) | 0.007 (0.007) | 0.006 (0.006) | 0.008 (0.006) |

Table S24: Predictive performance of PLANN extended for different activations functions with 1000 patients and 61% censoring-1. The standard deviation based on 1000 datasets is provided in parentheses.
